# Inflammatory ER stress responses dictate the immunopathogenic progression of systemic candidiasis

**DOI:** 10.1172/JCI167359

**Published:** 2023-09-01

**Authors:** Deepika Awasthi, Sahil Chopra, Byuri A. Cho, Alexander Emmanuelli, Tito A. Sandoval, Sung-Min Hwang, Chang-Suk Chae, Camilla Salvagno, Chen Tan, Liliana Vasquez-Urbina, Jose J. Fernandez Rodriguez, Sara F. Santagostino, Takao Iwawaki, E. Alfonso Romero-Sandoval, Mariano Sanchez Crespo, Diana K. Morales, Iliyan D. Iliev, Tobias M. Hohl, Juan R. Cubillos-Ruiz

**Affiliations:** 1 Department of Obstetrics and Gynecology, and; 2 Weill Cornell Graduate School of Medical Sciences, Weill Cornell Medicine, New York, New York, USA.; 3 Precision Immunology Institute, Icahn School of Medicine at Mount Sinai, New York, New York, USA.; 4 Unit of Excellence, Institute of Biology and Molecular Genetics, CSIC–Universidad de Valladolid, Valladolid, Spain.; 5 Laboratory of Comparative Pathology, Memorial Sloan Kettering Cancer Center, The Rockefeller University, and Weill Cornell Medicine, New York, New York, USA.; 6 Division of Cell Medicine, Medical Research Institute, Kanazawa Medical University, Ishikawa, Japan.; 7 Department of Anesthesiology, Pain Mechanisms Laboratory, Wake Forest University School of Medicine, Winston-Salem, North Carolina, USA.; 8 Department of Medicine and; 9 The Jill Roberts Institute for Research in Inflammatory Bowel Disease, Weill Cornell Medicine, New York, New York, USA.; 10 Infectious Disease Service, Department of Medicine, Memorial Sloan Kettering Cancer Center, New York, New York, USA.

**Keywords:** Immunology, Infectious disease, Cell stress, Fungal infections, Innate immunity

## Abstract

Recognition of pathogen-associated molecular patterns can trigger the inositol-requiring enzyme 1 α (IRE1α) arm of the endoplasmic reticulum (ER) stress response in innate immune cells. This process maintains ER homeostasis and also coordinates diverse immunomodulatory programs during bacterial and viral infections. However, the role of innate IRE1α signaling in response to fungal pathogens remains elusive. Here, we report that systemic infection with the human opportunistic fungal pathogen *Candida albicans* induced proinflammatory IRE1α hyperactivation in myeloid cells that led to fatal kidney immunopathology. Mechanistically, simultaneous activation of the TLR/IL-1R adaptor protein MyD88 and the C-type lectin receptor dectin-1 by *C*. *albicans* induced NADPH oxidase–driven generation of ROS, which caused ER stress and IRE1α-dependent overexpression of key inflammatory mediators such as IL-1β, IL-6, chemokine (C-C motif) ligand 5 (CCL5), prostaglandin E2 (PGE_2_), and TNF-α. Selective ablation of IRE1α in leukocytes, or treatment with an IRE1α pharmacological inhibitor, mitigated kidney inflammation and prolonged the survival of mice with systemic *C*. *albicans* infection. Therefore, controlling IRE1α hyperactivation may be useful for impeding the immunopathogenic progression of disseminated candidiasis.

## Graphical abstract

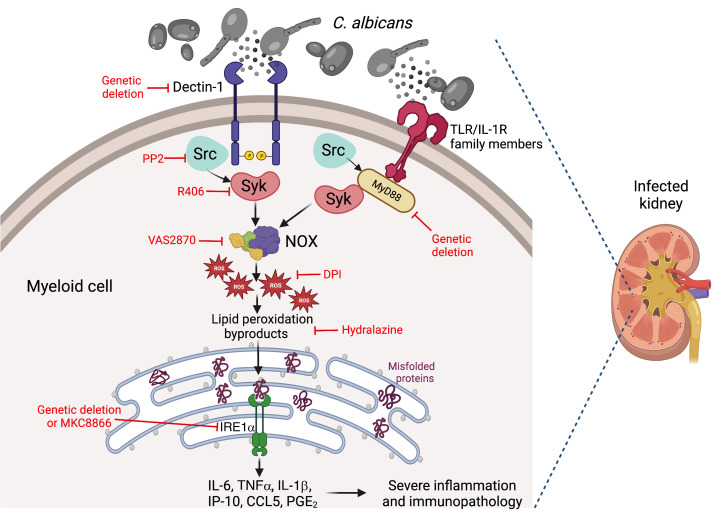

## Introduction

Sensing and responding to pathogens entails massive protein synthesis, folding, modification, and trafficking in immune cells, which are processes governed by the endoplasmic reticulum (ER). The excess demand in protein handling can lead to the accumulation of misfolded proteins in this organelle (1). Pathogens can also produce factors that alter the protein-folding capacity of the ER (2). Both events can provoke “ER stress,” a cellular state that activates the unfolded protein response (UPR) (1). Three ER stress sensors govern the UPR: inositol-requiring enzyme 1 α (IRE1α), protein kinase R–like endoplasmic reticulum kinase (PERK), and activating transcription factor 6 (ATF6). IRE1α is the most conserved ER stress sensor (1). When ER homeostasis is altered, IRE1α undergoes activation, prompting excision of a 26-nucleotide fragment from the *XBP1* mRNA. The spliced isoform generated (*XBP1s*) codes for the functionally active transcription factor XBP1, which activates various mechanisms to restore ER proteostasis (1). The canonical function of IRE1α-XBP1 in the UPR is to promote the expression of protein chaperones, foldases, glycosylases, quality control proteins, and ER-associated degradation components to restore ER proteostasis (1). However, concomitant with these adaptive responses, IRE1α signaling can also govern UPR-independent processes and pathways implicated in pathological conditions (3–6). Infection by bacterial and viral pathogens has been shown to activate the IRE1α branch of the UPR in immune and epithelial cells, which modulates host responses and disease progression (7). Our group further uncovered that IREα signaling drives the inducible expression of *Ptgs2* (Cox-2) and *Ptges* (m-PGES-1) in myeloid cells, promoting prostaglandin biosynthesis and behavioral pain responses in mice (8).

During infection, the interplay between host and pathogen factors determines the disease outcome (9). Inflammation plays a central role in this relationship and can dictate host survival by affecting the balance between protection and uncontrolled tissue damage (10). IRE1α activation has been shown to promote inflammation in pathophysiological conditions by driving the production of cytokines such as IL-1β, IL-6, IL-23, and TNF-α (11–15). However, the role of immune-intrinsic IRE1α signaling in fungal infections is unknown.

Dectin-1 is a C-type lectin receptor that recognizes pathogen-associated molecular patterns (PAMPs) such as β-glucans in fungal cell walls (16, 17). Upon binding, the cytoplasmic immunoreceptor tyrosine-based activation motif (ITAM) of dectin-1 is phosphorylated by the Src family of kinases, providing a docking site for the spleen tyrosine kinase (Syk). Syk subsequently activates protein kinase C-δ (PKCδ) to mediate phosphorylation of the adaptor protein CARD9. This enables CARD9 to complex with BCL10 and MALT1 to activate κβ-dependent transactivation (17–19). Syk also relays signals to Vav-PLCγ2, thus activating the NADPH oxidase (NOX) to produce abundant ROS (20–22) that mediate fungal killing (23, 24). Importantly, *C*. *albicans* β-glucans further activate TLR/IL-1R–MyD88 signaling in myeloid cells, which exacerbates intracellular ROS production (25). Recognition of β-glucans by these pathways induces innate effector functions, including phagocytosis, biosynthesis of lipid mediators, and expression of cytokines and chemokines such as IL-6, TNF-α, IL-1β, C-X-C motif chemokine ligand 1 (CXCL1), and C-X-C motif chemokine ligand 2 (CXCL2), among others (26). Here, we identify a mechanism whereby activation of MyD88 and dectin-1 by *C*. *albicans* triggered the IRE1α arm of the UPR in myeloid cells via Src-Syk-NOX activation, ROS production, and the generation of lipid peroxidation byproducts.

*Candida albicans*, a commensal fungus, is a human opportunistic pathogen that causes local mucosal infections as well as life-threatening bloodstream systemic infections (27). Intravenous *C*. *albicans* challenge in mice has been extensively used to model clinical disseminated infection (28). In this system, *C*. *albicans* is detected in the brain and kidney after entering the bloodstream, which similarly occurs in humans (29, 30). The high blood flow received by the kidneys, which reaches 25% of cardiac output, makes this organ a distinctive niche for fungal proliferation, leading to massive recruitment of innate immune cells that are aimed at controlling the infection at this location (28, 31, 32). Nonetheless, unrestrained recruitment of myeloid cells to the infected kidney and the excessive production of inflammatory mediators that accompany this process often cause immune-driven pathology, leading to kidney tissue damage, sepsis, and death (31–33). Accordingly, deletion of the chemokine receptor CCR1 or the type 1 interferon receptor (IFNAR-1) can reduce neutrophil-driven kidney immunopathology without altering fungal burden in this organ, increasing the survival of mice with candidemia (34, 35)

Understanding the immunopathogenic mechanisms of disseminated *C*. *albicans* infection is an area of active investigation, but whether immune-intrinsic ER stress responses participate in this deadly process has not been established. The present study reveals that activation of the ER stress sensor IRE1α in myeloid cells promoted the fatal progression of systemic candidiasis in mice. Genetic or pharmacological abrogation of IRE1α reduced kidney immunopathology and extended host survival, suggesting that targeting this pathway might represent a plausible treatment approach for this disease.

## Results

### Systemic C. albicans infection triggers IRE1α in kidney-infiltrating immune cells.

**Figure 1:**
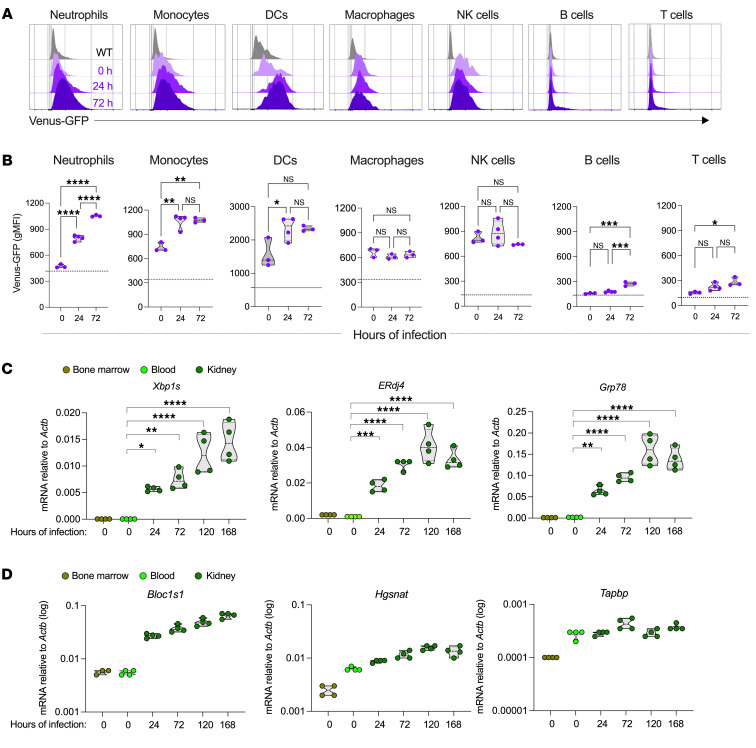
Systemic *C*. *albicans* infection enhances IRE1α activation in kidney-resident myeloid cells. (**A** and **B**) ERAI or WT C57BL/6J mice (*n* = 3–4 per time point) were left untreated or injected i.v. with 10^5^ *C*. *albicans* cells. Venus reporter expression was assessed by flow cytometry in diverse immune cells infiltrating the kidney at the indicated time points of infection. (**A**) Representative histograms depicting Venus levels in immune cells over time. Gray histograms indicate WT mice that do not express the reporter. Purple histograms denote ERAI mice. (**B**) Geometric mean fluorescence intensity (gMFI) of Venus expression in the indicated immune cell populations and times. Dashed lines represent intrinsic autofluorescence in WT mice. (**C** and **D**) WT C57BL/6J mice (*n* = 4 per time point) were injected i.v. with 10^5^ *C*. *albicans* cells, and kidney-infiltrating Ly6G^+^ neutrophils were isolated on days 1, 3, 5, and 7 after infection. As comparative controls, Ly6G^+^ neutrophils were purified from the blood and bone marrow of naive mice (*n* = 3–4). Expression of the indicated transcripts was determined by RT-qPCR and normalized to endogenous *Actb* in each sample. (**C**) mRNA expression of UPR-related genes and (**D**) RIDD target genes. Data are shown as the mean ± SEM. *P < 0.05, **P < 0.005, ***P < 0.0005, and ****P < 0.0001, by 1-way ANOVA with Tukey’s test (**B**) and 2-way ANOVA with Dunnett’s test (**C**).

Myeloid cell activation and accumulation dictate the balance between protective and pathological innate immune responses during candidiasis (25, 31, 35–37), but the involvement of the UPR in controlling this balance remains elusive. We evaluated whether systemic infection with *C*. *albicans* elicited IRE1α activation and the UPR in immune cells in vivo. To this end, we used ER stress–activated indicator (ERAI) mice that express the Venus fluorescent protein in cells undergoing IRE1α activation (38). C57BL/6J WT and ERAI mice were i.v. challenged with 10^5^ *C*. *albicans* SC5314 yeast cells*,* and their kidneys, blood, spleen, and bone marrow were analyzed at multiple time points thereafter. Consistent with prior reports (35, 36), we found that systemic *C*. *albicans* infection provoked drastic changes in the kidney immune contexture, wherein the proportion of neutrophils and monocytes increased significantly 24 and 72 hours after infection (Supplemental Figure 1, A–C; supplemental material available online with this article; https://doi.org/10.1172/JCI167359DS1). These alterations were accompanied by reduced proportions of macrophages, T cells, and B cells in the kidney after infection, while DCs and NK cells remained unchanged (Supplemental Figure 1, A–C). No differences in immune cell infiltration were observed when C57BL/6J WT or ERAI mice were used as infection hosts (Supplemental Figure 1, B and C). The non-leukocyte compartment of the kidney, which encompasses multiple types of epithelial cells, did not demonstrate major alterations in IRE1α activation upon infection (Supplemental Figure 1D). Notably, neutrophils in the kidneys of mice with candidemia demonstrated rapid and progressive IRE1α activation, as evidenced by a time-dependent increase in their Venus reporter signal, compared with kidney-resident neutrophils from naive mice (Figure 1, A and B). Blood and splenic neutrophils showed modest IRE1α activation 72 hours after infection, whereas negligible changes were observed in this population in the bone marrow (Supplemental Figure 1, E–G). Kidney-resident monocytes and DCs in naive mice demonstrated strong constitutive IRE1α activation, which increased 24 hours after *C*. *albicans* infection and remained steady thereafter (Figure 1, A and B). Monocytes and DCs at other locations showed minimal changes in IRE1α activation after infection compared with their naive counterparts (Supplemental Figure 1, E–G). Furthermore, systemic challenge with *C*. *albicans* did not alter IRE1α activation in macrophages, NK cells, T cells, or B cells in the kidneys, blood, spleen, or bone marrow at the time points analyzed (Figure 1, A and B, and Supplemental Figure 1, E–G). Therefore, enhanced IRE1α activity was predominantly detected in neutrophils, monocytes, and DCs infiltrating the kidney upon *C*. *albicans* systemic infection. To confirm these observations, we isolated kidney-resident neutrophils at multiple postinfection time points and assessed the expression of canonical UPR marker genes. We detected high levels of *Xbp1s*, *ERdj4*, and *Grp78* in kidney-infiltrating neutrophils 24 hours after i.v. challenge with *C*. *albicans*, and the relative abundance of these genes drastically increased in a time-dependent manner (Figure 1C). In these experiments, neutrophils isolated from blood and bone marrow of naive mice were used as comparative controls, since we were unable to isolate a sufficient number of neutrophils from the kidneys of naive mice. Despite marked IRE1α and UPR activation in kidney-infiltrating neutrophils from infected mice (Figure 1C), we did not observe regulated IRE1α-dependent decay (RIDD) in these cells, as the levels of typical RIDD target genes did not decrease throughout disease progression (Figure 1D). These data reveal that systemic candidiasis induced pronounced IRE1α activation in multiple myeloid cell subsets that infiltrated the infected kidneys.

### Dectin-1 and MyD88 signaling induces IRE1α activation via the Src-Syk-NOX pathway.

**Figure 2:**
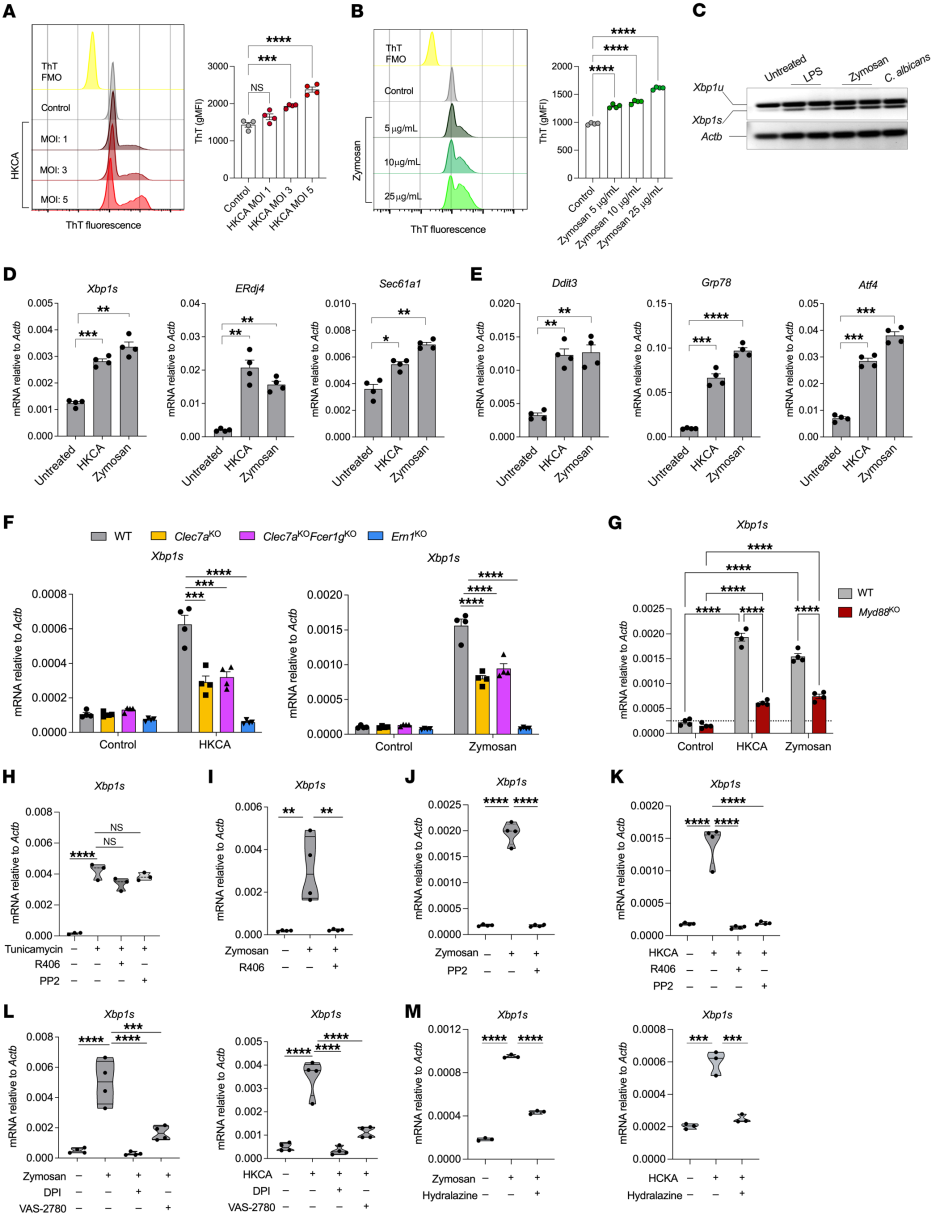
Molecular pathways mediating IRE1α activation in *C. albicans*–exposed neutrophils. (**A** and **B**) Bone marrow–resident neutrophils isolated from WT C57BL/6J mice (*n* = 4) were stimulated with HKCA or zymosan for 6 hours as indicated. Intracellular protein aggregates were assessed via ThT staining. Representative histograms and bar graphs for HKCA (**A**) and zymosan (**B**) are shown. (**C**) Neutrophils were exposed to vehicle control (Untreated), LPS (100 ng/mL), zymosan (25 μg/mL), or live *C*. *albicans* (MOI = 10) for 6 hours, and *Xbp1* splicing was assessed by conventional RT-PCR. *Xbp1u*, unspliced form; *Xbp1s*, spliced form. Data are representative of at least 3 independent experiments with similar results. (**D** and **E**) Bone marrow neutrophils from WT C57BL/6J mice (*n* = 4) were stimulated with HKCA (MOI = 1) or zymosan (25 μg/mL) for 6 hours, and expression of the indicated transcripts was determined by RT-qPCR. (**F** and **G**) Bone marrow–resident neutrophils were isolated from mice of the indicated genotypes (*n* = 4/genotype) and stimulated for 6 hours with HKCA (MOI = 10) or zymosan (25 μg/mL). (**H**–**M**) Bone marrow–resident neutrophils from WT C57BL/6J mice (*n* = 3–4) were pretreated with the indicated inhibitors and stimulated for 6 hours as described in Methods. (**F**–**M**) *Xbp1s* levels were determined using RT-qPCR, and data were normalized to endogenous *Actb*. Data are shown as the mean ± SEM (**A**–**G**) or violin plots (**H**–**M**). Data are representative of 3–4 independent experiments with similar results. *P < 0.05, **P < 0.005, ***P < 0.0005, and ****P < 0.0001, by 1-way ANOVA with Dunnett’s multiple-comparison test (**A** and **B**), 2-tailed Student’s *t* test (**D** and **E**), 2-way ANOVA with Šídák’s multiple-comparison test (**G**), and 1-way ANOVA with Tukey’s test (**F** and **H**–**M**).

We sought to define how *C*. *albicans* triggers ER stress responses in myeloid cells. ER stress and UPR activation normally occur as a result of the accumulation of misfolded or unfolded proteins in this organelle (1). Hence, neutrophils isolated from the bone marrow of naive mice were stimulated ex vivo with zymosan — a fungal β-glucan recognized by dectin-1 and surface TLRs — or with heat-killed *C*. *albicans* (HKCA), and the intracellular accumulation of misfolded protein aggregates was evaluated using thioflavin T (ThT) staining (39, 40). We found a dose-dependent increase in the levels of ThT^+^ aggregates in primary neutrophils exposed to either zymosan or HKCA (Figure 2, A and B). Accordingly, neutrophils exposed to zymosan or live *C*. *albicans* also demonstrated robust IRE1α-dependent splicing of *Xbp1* mRNA (Figure 2C). In this assay, bacterial LPS was used as a positive control, since this TLR4 agonist has been shown to trigger IRE1α-XBP1 in myeloid leukocytes (8, 12). Real-time quantitative PCR (RT-qPCR) analyses further confirmed that neutrophils exposed to either zymosan or HKCA overexpressed the *Xbp1s* isoform, while markedly upregulating canonical IRE1α/XBP1 target genes such as *Sec61a1* and *ERdj4* (41) (Figure 2D), as well as the global UPR gene markers *Ddit3*, *Grp78*, and *Atf4* (Figure 2E). Hence, zymosan or *C*. *albicans* stimulation directly caused ER stress and triggered the UPR in primary neutrophils.

Dectin-1, encoded by *Clec7a*, is a major C-type lectin receptor (CLR) for β-glucans that enables innate recognition of fungi by myeloid cells (42–44). To determine whether activation of IRE1α upon exposure to *C*. *albicans* yeast cells was mediated by this CLR, we isolated bone marrow–resident neutrophils from WT or *Clec7a*-knockout (*Clec7a^KO^*) mice and then stimulated the cells with HKCA or zymosan. We also used IRE1α-deficient (*Ern1^KO^*) neutrophils as a control for *Xbp1s* detection specificity. Of note, dectin-1–deficient neutrophils exposed to either zymosan or HKCA demonstrated impaired IRE1α activation, as evidenced by an approximately 50% reduction in the levels of *Xbp1s* transcripts generated in comparison with their WT counterparts (Figure 2F), and similar results were observed when bone marrow–resident monocytes isolated from *Clec7a^KO^* mice were analyzed (Supplemental Figure 2A). Additional CLRs such as dectin-2, dectin-3, and Mincle can recognize fungal products and induce intracellular signaling via interaction with Fc receptor γ (FcRγ) chain–associated ITAMs (45). Thus, to discern whether engagement of these CLRs might contribute to IRE1α activation by *C*. *albicans*, we tested neutrophils that simultaneously lacked both dectin-1 and FcRγ (*Clec7a^KO^* *Fcer1g^KO^*). We observed a similar decrease in *Xbp1s* levels in dectin-1–KO and dectin-1/FcRγ double-KO neutrophils stimulated with either HKCA or zymosan, compared with expression levels in their WT counterparts (Figure 2F). The residual IRE1α activation observed upon concomitant loss of dectin-1 and FcRγ suggested that engagement of other PRRs, likely TLRs, could be implicated. Indeed, zymosan β-glucans activate dectin-1 and TLR2, whereas *C*. *albicans* wall products, such as β-glucans and mannans, can simultaneously trigger TLR2, TLR4, and dectin-1 and induce IL-1α and IL-1β release by innate immune cells (25, 46, 47). We used MyD88-deficient neutrophils to evaluate the contribution of TLR2/-4 and IL-1R signaling in this process. Of note, IRE1α activation in zymosan- or HKCA-treated neutrophils lacking MyD88 was reduced by approximately 65% (Figure 2G). These data indicate that both dectin-1 and MyD88 signaling mediated maximal IRE1α activation in neutrophils exposed to zymosan or *C*. *albicans*.

The ITAM of dectin-1 causes Syk activation via Src, and this process induces downstream effector functions such as pathogen killing and cytokine production (17–19). Similarly, engagement of membrane-bound TLRs has been proposed to trigger rapid Rac1-driven activation of Src, which in turn activates the Syk-MyD88 molecular complex by phosphorylating MyD88 (48). We used the selective Src inhibitor PP2 (49, 50) and the Syk inhibitor R406 (51, 52) to evaluate whether these kinases mediate IRE1α activation in neutrophils sensing β-glucans or *C*. *albicans*. Importantly, while PP2 or R406 did not compromise IRE1α activation in response to pharmacological ER stress caused by tunicamycin treatment (Figure 2H), these inhibitors completely abrogated *Xbp1* splicing in neutrophils (Figure 2, I–K) and monocytes (Supplemental Figure 2B) exposed to zymosan or HKCA. Hence, the Src-Syk axis was necessary for optimal IRE1α activation in *C*. *albicans*–exposed neutrophils via upstream dectin-1 and TLR/IL-1R–MyD88 signaling inputs.

Upon *C*. *albicans* or zymosan exposure, Syk activation induces ROS generation via NOX (17, 53, 54). Importantly, ROS accumulation can cause ER stress and IRE1α activation by promoting the generation of lipid peroxidation byproducts, such as 4-HNE, that modify ER-resident chaperones and disrupt protein folding in this organelle (55, 56). We tested whether NOX-derived ROS could drive IRE1α activation in neutrophils exposed to *C*. *albicans*. Bone marrow–resident neutrophils stimulated ex vivo with either zymosan or HKCA demonstrated copious ROS production, as indicated by a rapid conversion of 2′,7′–dichlorofluorescein diacetate (DCFDA) to its oxidized fluorescent form, 2′,7′–dichlorofluorescein (DCF) (57) (Supplemental Figure 2, C and D). Treatment with either diphenyleneiodonium (DPI), an inhibitor of both nitric oxide synthase and NOX (58), or VAS-2870, a NOX-specific inhibitor (59), suppressed ROS generation (Supplemental Figure 2, C and D) and inhibited IRE1α-dependent *Xbp1* splicing (Figure 2L) in neutrophils stimulated with zymosan or HKCA. Treatment with Mito-TEMPO or MitoQ , which specifically scavenge mitochondria-derived ROS (60, 61), did not affect this process (Supplemental Figure 2E), indicating that NOX-derived ROS are predominant inducers of IRE1α activation in neutrophils sensing *C*. *albicans*. Sequestering lipid peroxidation byproducts with the hydrazine derivative hydralazine (55, 56) abrogated IRE1α activation in neutrophils exposed to zymosan or HKCA (Figure 2M). Importantly, treatment with VAS-2870 and hydralazine also prevented IRE1α activation in monocytes sensing zymosan or *C*. *albicans* (Supplemental Figure 2F). Hence, engagement of dectin-1 and MyD88 in *C*. *albicans*–exposed myeloid cells promoted overproduction of NOX-dependent ROS and lipid peroxidation byproducts that activated IRE1α.

### Selective deletion of IRE1α in leukocytes increases the survival of C. albicans–infected hosts.

**Figure 3:**
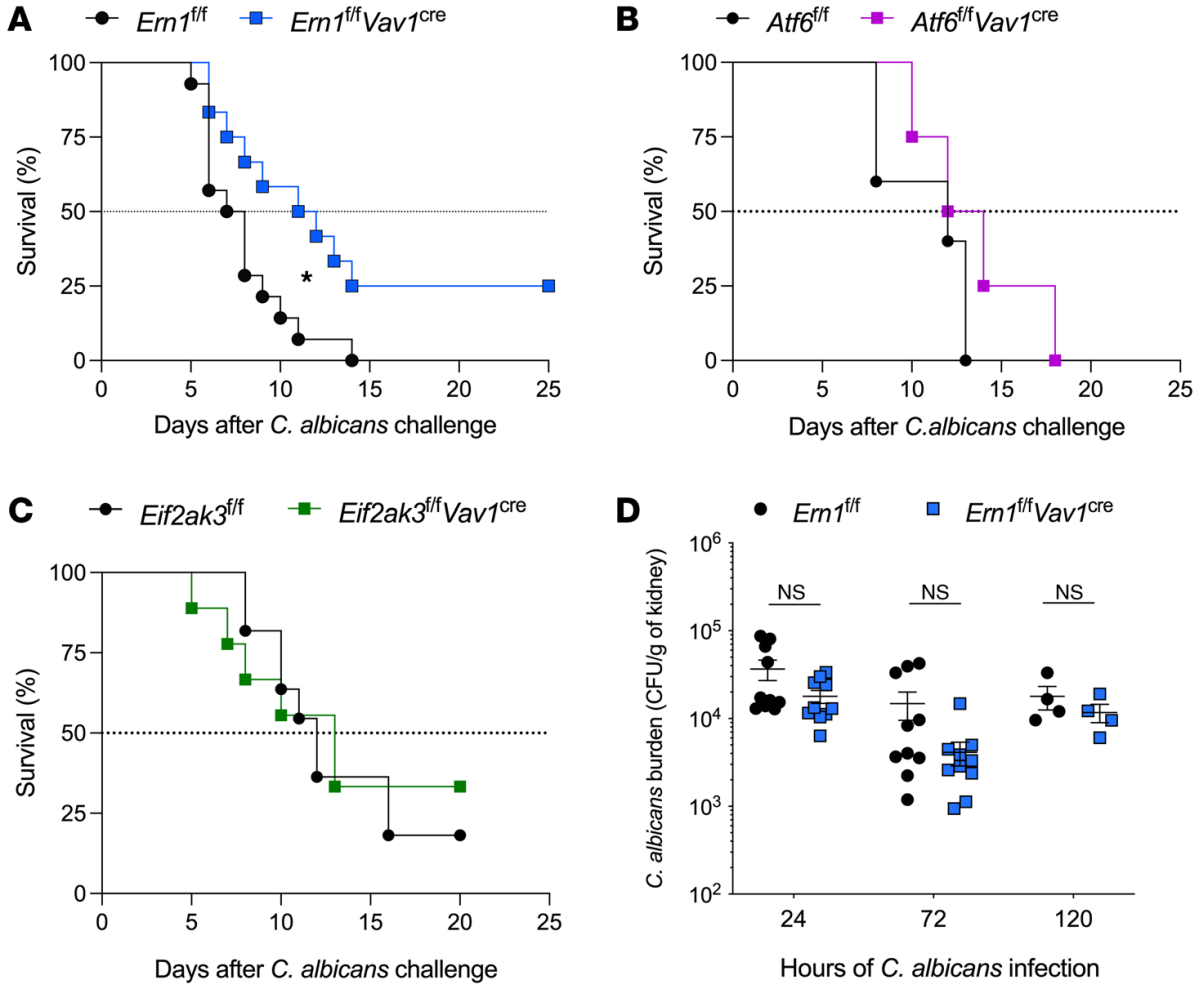
Loss of IRE1α in leukocytes increases survival in mice with systemic candidiasis. (**A**–**C**) Mice of the indicated genotypes were infected i.v. with 10^5^ *C*. *albicans,* and host survival was monitored over time. (**A**) *Ern1^fl/fl^* (*n* = 14) or *Ern1^fl/fl^* *Vav1^Cre^* (*n* = 12) mice, (**B**) *Atf6^fl/fl^* (*n* = 5) or *Atf6^fl/fl^* *Vav1^Cre^* (*n* = 4) mice, (**C**) *Eif2ak3^fl/fl^* (*n* = 11) or *Eif2ak3^fl/fl^* *Vav1^Cre^* (*n* = 9) mice, and (**D**) *Ern1^fl/fl^* or *Ern1^fl/fl^* *Vav1^Cre^* mice (*n* = 4–10 mice of each genotype per time point) were infected i.v. with 10^5^ *C*. *albicans,* and fungal burden in the kidney was determined as CFU per gram of tissue at the indicated time points after infection. *P < 0.05, by Student’s *t* test, by log-rank rest (**A**) and Student’s *t* test (**D**).

We sought to define the functional role of immune-intrinsic IRE1α during systemic candidiasis. To this end, we used *Ern1^fl/fl^* *Vav1^Cre^*–transgenic mice, which have selective deletion of this ER stress sensor in the hematopoietic compartment (62, 63). *Ern1^fl/fl^* *Vav1^Cre^* mice, or their IRE1α-sufficient (*Ern1^fl/fl^*) littermate controls, were challenged i.v. with 10^5^ *C*. *albicans* SC5314 yeast cells, and overall host survival was monitored thereafter. Strikingly, loss of IRE1α in leukocytes extended host survival and led to a full recovery in approximately 25% of mice with disseminated candidiasis, compared with their IRE1α-sufficient (*Ern1^fl/fl^*) counterparts (Figure 3A). We observed a similar increase in survival when *Ern1^fl/fl^* *Mrp8^Cre^* mice, which have selective deletion of IRE1α in neutrophils (64), were used in these experiments (Supplemental Figure 3A), revealing that neutrophil-intrinsic IRE1α played a major role in the lethal progression of candidemia. Notably, deletion of other ER stress sensors, such as PERK or ATF6, did not affect the survival rates after *C*. *albicans* infection (Figure 3, B and C).

We next evaluated whether ablation of IREα in leukocytes increases host survival by reducing fungal burden in the kidney. Surprisingly, the number of *C*. *albicans* cells recovered from whole kidney homogenates was comparable in mice of both genotypes at multiple time points after infection (Figure 3D), suggesting that loss of IRE1α in leukocytes did not alter *C*. *albicans* killing in vivo. Indeed, IRE1α-deficient neutrophils, the main effector cells in this setting, showed no alterations in their phagocytic ability (Supplemental Figure 3B) or in their production of pathogen-killing mediators such as ROS, myeloperoxidase (MPO), or neutrophil extracellular traps (NETs) (Supplemental Figure 3, C–H). Accordingly, the *C*. *albicans*–killing capacity of IRE1α-deficient neutrophils was comparable to that of their WT counterparts (Supplemental Figure 3I). These data unveil that immune-intrinsic IRE1α signaling supported fatal disease progression in mice systemically infected with *C*. *albicans*.

### IRE1α promotes kidney tissue damage in mice with systemic C. albicans infection.

**Figure 4:**
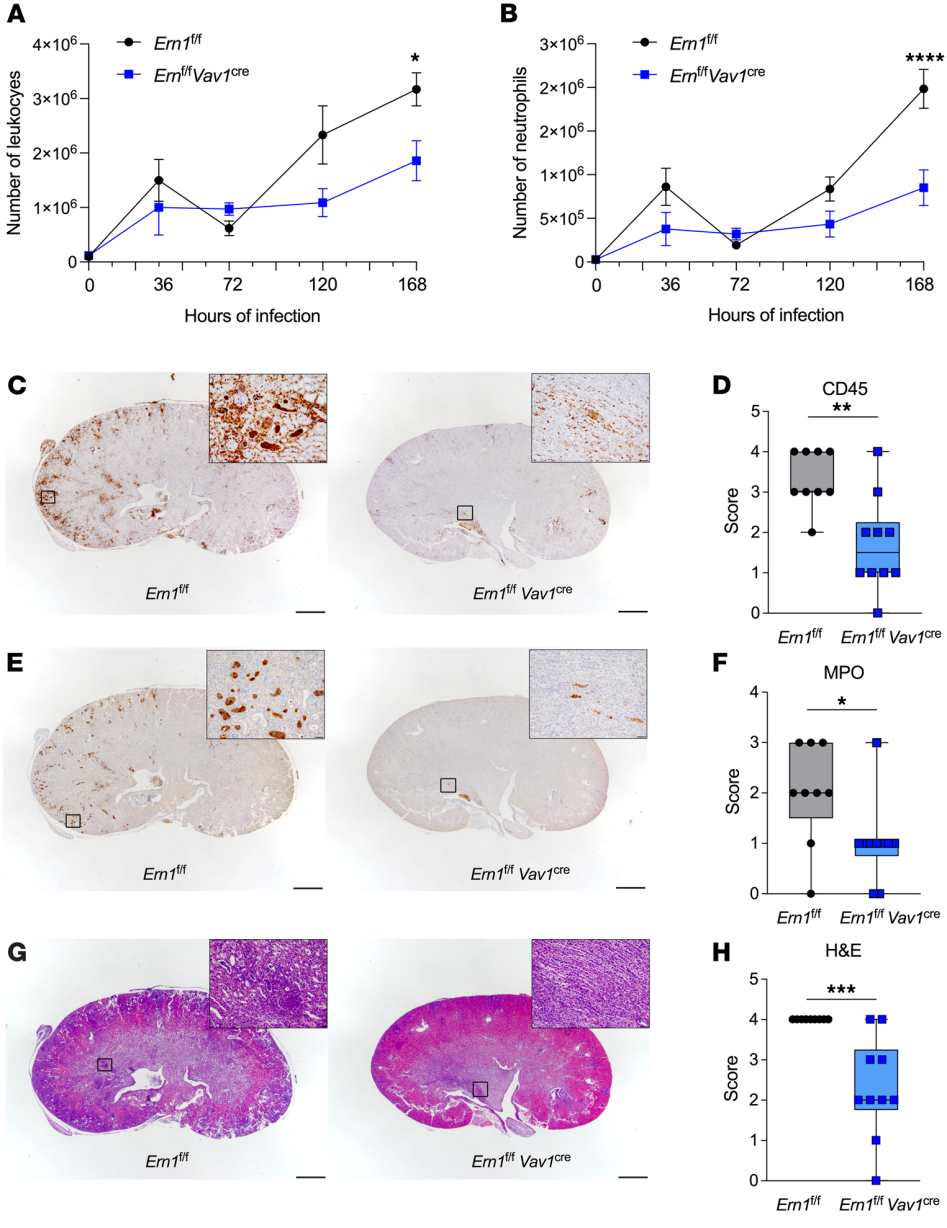
IRE1α deficiency reduces kidney tissue damage in mice with systemic candidiasis. (**A** and **B**) *Ern1^fl/fl^* or *Ern1^fl/fl^* *Vav1^Cre^* mice (*n* = 3–4 per genotype per time point) were challenged i.v. with 10^5^ *C*. *albicans* cells, and the numbers of CD45^+^ leukocytes (**A**) and CD45^+^CD11b^+^Ly6G^+^Ly6C^lo^ neutrophils (**B**) in their kidneys were determined by flow cytometry at the indicated time points of infection. (**C**–**H**) *Ern1^fl/fl^* (*n* = 9) or *Ern1^fl/fl^* *Vav1^Cre^* (*n* = 10) mice were infected i.v. with 10^5^ *C*. *albicans*, and kidney sections were stained 7 days later for CD45 (**C** and, **D**), MPO (**E** and **F**), and H&E (**G** and **H**). Representative images of kidney section staining (**C**, **E**, and **G**) and their corresponding pathological scoring (**D**, **F**, and **H**) are shown. Scale bars: 1 mm; 50 μm (insets). *P < 0.05 and ****P < 0.0001, by 2-way ANOVA with Šídák’s multiple-comparison test (**A** and **B**). Data in **D**, **F**, and **H** are presented as the median plus lower and higher quartiles (boxes), with minimum and maximum values (whiskers). *P < 0.05, **P < 0.005, and ***P < 0.0005, by Mann-Whitney *U* test (**D**, **F**, and **H**)

After gaining access to the bloodstream, *C*. *albicans* can reach the glomeruli and penetrate the renal interstitium, eliciting an influx of inflammatory myeloid cells capable of causing severe immunopathology and lethal kidney damage (30, 34, 35). Thus, we next determined the contribution of IRE1α activation to the immunopathogenesis of invasive candidiasis. *Ern1^fl/fl^* *Vav1^Cre^* mice, or their IRE1α-sufficient (*Ern1^fl/fl^*) littermate controls, were challenged i.v. with 10^5^ *C*. *albicans* SC5314 yeast cells, and their kidneys were resected at different postinfection time points for histopathological and immunophenotyping analyses. We found that loss of IRE1α in hematopoietic cells did not alter kidney infiltration by monocytes, macrophages, B cells, or NK cells after *C*. *albicans* challenge (Supplemental Figure 4, A–D). A modest reduction in T cell and DC infiltration was observed in the kidneys of *Ern1^fl/fl^* *Vav1^Cre^* mice 5 days after infection, but comparable numbers of these leukocyte subsets were found thereafter in hosts of both genotypes (Supplemental Figure 4, E and F). The number of total leukocytes and CD45^+^CD11b^+^Ly6G^+^ neutrophils in the kidneys was similar in mice of either genotype before infection and 1, 3, and 5 days after *C*. *albicans* challenge (Figure 4, A and B). Yet, we found a reduced number of CD45^+^CD11b^+^Ly6G^+^ neutrophils in the kidneys of *Ern1^fl/fl^* *Vav1^Cre^* mice systemically infected with *C*. *albicans* for 7 days, compared with their IRE1α-sufficient counterparts (Figure 4, A and B). Histopathological analyses of kidney sections at this advanced time point confirmed decreased neutrophilic infiltration in *Ern1^fl/fl^* *Vav1^Cre^* mice, as determined by parallel staining for CD45 and MPO (Figure 4, C–F). Most important, H&E staining of the same kidney sections demonstrated that loss of IRE1α in leukocytes significantly diminished the extent of coalescing inflammatory foci, while reducing the amount of degenerative and fibrotic lesions, compared with their IRE1α-sufficient counterparts (Figure 4, G and H). Hence, immune-intrinsic IRE1α promoted kidney tissue damage in mice with disseminated candidiasis.

### IRE1α activation drives overt kidney inflammation in hosts with disseminated candidiasis.

**Figure 5:**
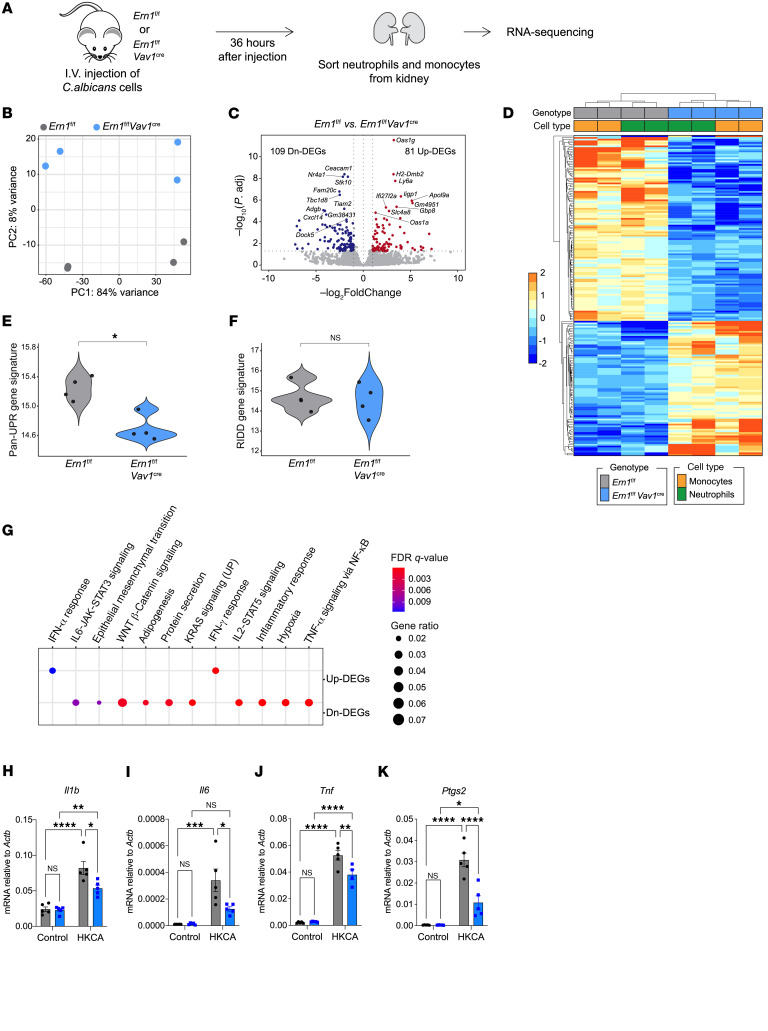
Gene expression profiles controlled by IRE1α in kidney-infiltrating neutrophils and monocytes from mice with systemic candidiasis. (**A**–**G**) *Ern1^fl/fl^* or *Ern1^fl/fl^* *Vav1^Cre^* mice were infected with 10^5^ *C*. *albicans* cells, and 36 hours later, kidney-infiltrating neutrophils (Ly6G^+^Ly6C^lo^) and monocytes (Ly6C^hi^) were sorted (*n* = 4 per genotype) for RNA-Seq analyses. (**A**) Experimental scheme. (**B**) PCA showing distinct clustering of each genotype. (**C**) Volcano plot highlighting the top 10 upregulated (Up) and downregulated (Dn) DEGs. The 2 vertical dashed lines on each side correspond to –1.0 and 1.0 cut points, which are log_2_ fold-change cutoffs used for determining DEGs. The horizontal dashed line corresponds to *P*-adjusted (P.adj) values of 0.05, which was another cutoff used for determining DEGs. (**D**) Heatmap displaying the 81 upregulated and 109 downregulated DEGs in *Ern1^fl/fl^* *Vav1^Cre^* compared with *Ern1^fl/fl^* cells. (**E** and **F**) Pathway score analyses showing significant downregulation of global UPR gene markers in (**E**) *Ern1^fl/fl^* *Vav1^Cre^* neutrophils and monocytes compared with their *Ern1^fl/fl^* counterparts, (**F**) whereas no significant difference was observed for RIDD target genes. *P = 0.029, by Wilcoxon test. (**G**) Pathway enrichment analysis depicting significant downregulation of multiple inflammatory gene programs. (**H**–**K**) *Ern1^WT^* (gray bars) or *Ern1^KO^* (blue bars) neutrophils isolated from the bone marrow (*n* = 4–5 per genotype) were stimulated with HKCA (MOI = 10) for 6 hours, and expression of the indicated transcripts was determined by RT-qPCR. *P < 0.05, **P < 0.005, ***P < 0.0005, and ****P < 0.0001, by 2-way ANOVA with Šídák’s multiple-comparison test (**H**, **I**, and **K**) and 2-way ANOVA with Tukey’s multiple-comparison test (**J**).

To further understand the detrimental effects of innate IRE1α signaling during systemic *C*. *albicans* infection, we performed transcriptomic analyses of neutrophils and monocytes sorted from the kidneys of *Ern1^fl/fl^* or *Ern1^fl/fl^* *Vav1^Cre^* mice 36 hours after *C*. *albicans* i.v. challenge (Figure 5A). Principal component analysis (PCA) showed a distinct separation of global gene expression profiles based on the presence or absence of IRE1α (Figure 5B). Among 190 differentially expressed genes (DEGs), 109 were downregulated and 81 were upregulated in IRE1α-deficient monocytes and neutrophils compared with their IRE1α-sufficient counterparts (Figure 5, C and D, and Supplemental Table 1). Loss of IRE1α led to decreased expression of ER stress sensor–induced UPR genes, such as *Edem1*, *Sec61a1*, *Dnajb9*, and *Hspa5*, but did not alter the levels of typical RIDD target genes (Figure 5, E and F). Notably, downstream pathway analysis using Hallmark gene sets from the Molecular Signatures Database (MSigDB) revealed significant downregulation of transcriptional network genes associated with inflammatory responses, as well as of IL-6–JAK–STAT3, TNF-α, and IL-2–STAT5 signaling genes in IRE1α-deficient neutrophils and monocytes infiltrating the kidneys compared with their WT counterparts (Figure 5G). These data indicate that IRE1α activation in response to *C*. *albicans* caused transcriptional reprogramming of kidney-infiltrating myeloid cells toward a highly inflammatory state. Supporting this concept, we found that IRE1α-deficient neutrophils exposed to HKCA showed lower expression of classical proinflammatory genes such as *Il1b*, *Il6*, *Tnf*, and *Ptges2*/*Cox2* than did their IRE1α-sufficient counterparts (Figure 5, H–K).

**Figure 6:**
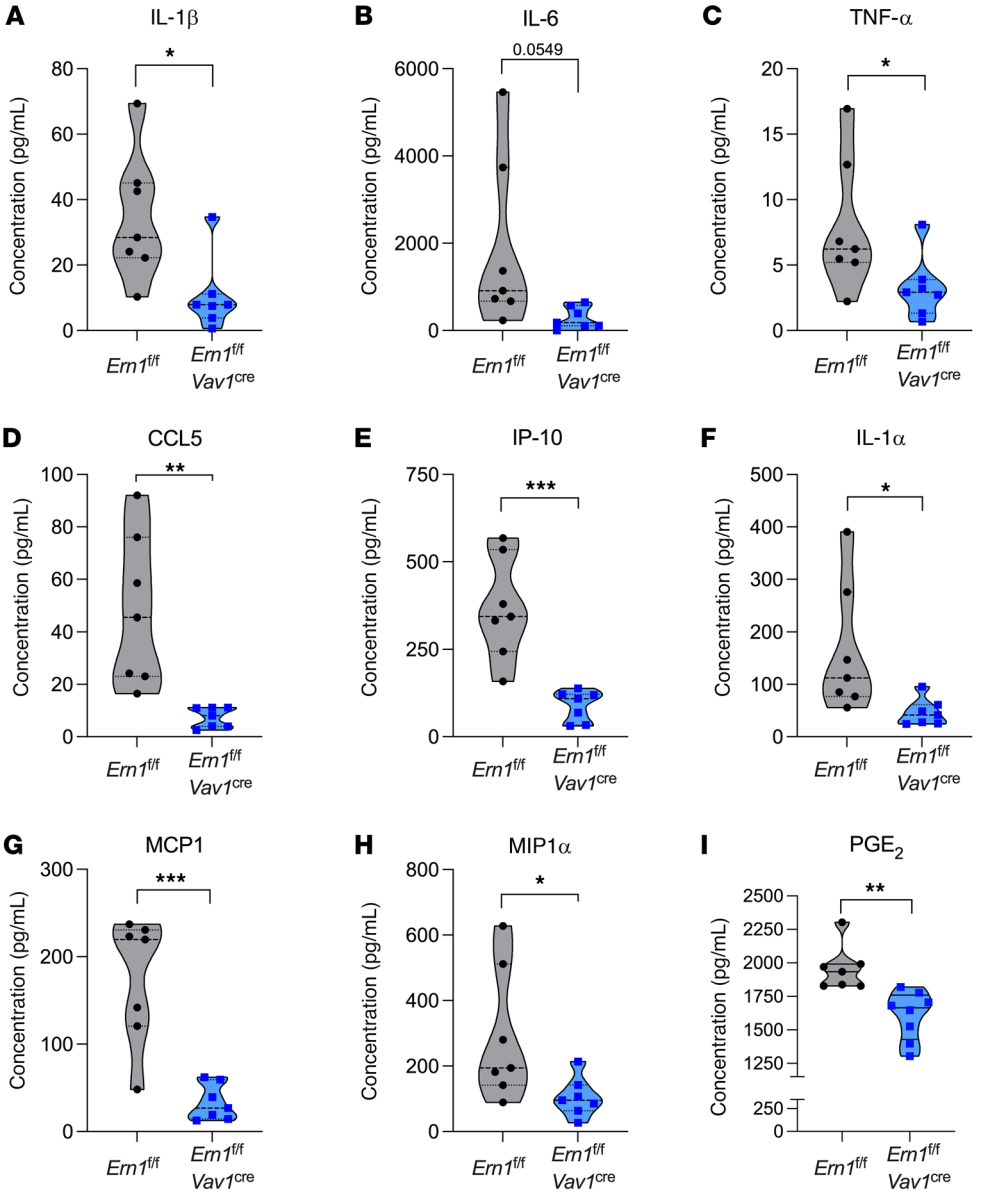
Decreased production of inflammatory factors in the kidneys of *C*. *albicans*–infected mice lacking IRE1α in leukocytes. (**A**–**I**) *Ern1^fl/fl^* or *Ern1^fl/fl^* *Vav1^Cre^* mice (*n* = 7–8 per group) were infected i.v. with 10^5^ *C*. *albicans* cells, and 3 days later, supernatants from whole kidney homogenates were analyzed for cytokine and chemokine levels (**A**–**H**), as well as PGE_2_ expression (**I**). Data are shown using violin plots. *P < 0.05, **P < 0.005, and ***P < 0.0005, by 2-tailed Student’s *t* test.

Beyond its canonical function in the UPR, IRE1α signaling can enhance the production of inflammatory mediators via diverse mechanisms (8, 12–14). In addition, high levels of IL-1β, IL-6, and TNF-α produced in the kidney at the initial stages of systemic *C*. *albicans* infection can contribute to the immunopathogenesis of the disease (28, 35, 65). We examined whether IRE1α overactivation in myeloid cells infiltrating the kidney upon i.v. *C*. *albicans* challenge promotes the local overexpression of factors contributing to renal inflammation and tissue damage. Thus, we conducted a comprehensive analysis of cytokines and chemokines in whole kidney samples from *Ern1^fl/fl^* and *Ern1^fl/fl^* *Vav1^Cre^* mice systemically infected with *C*. *albicans* for 3 days, which had a similar fungal burden (Figure 3D) and comparable immune cell infiltration (Figure 4, A and B, and Supplemental Figure 4, A–F). We found reduced levels of IL-1β, IL-6, and TNF-α in supernatants from kidney homogenates of *Ern1^fl/fl^* *Vav1^Cre^* mice, compared with their IRE1α-sufficient counterparts at the same time point (Figure 6, A–C). Additional inflammatory mediators such as and chemokine (C-C motif) ligand 5 (CCL5), IP-10 (also known as CXCL10), IL-1α, MCP-1 (also known as CCL2), MIP1α, prostaglandin E2 (PGE_2_) were also significantly decreased in the kidneys of infected *Ern1^fl/fl^* *Vav1^Cre^* mice at this time point (Figure 6, D–I), while other factors such as IFN-β, IL-10, eotaxin, IL-2, IL-12 p40, keratinocyte-derived cytokine (KC), and macrophage colony-stimulating factor (MCSF) remained unaltered (Supplemental Figure 5). Hence, genetic ablation of IRE1α in leukocytes restrained the early overexpression of major proinflammatory mediators in the kidneys, thereby mitigating the subsequent immune-driven damage to this organ.

### Therapeutic effects of IRE1α inhibition in mice with systemic candidiasis.

**Figure 7:**
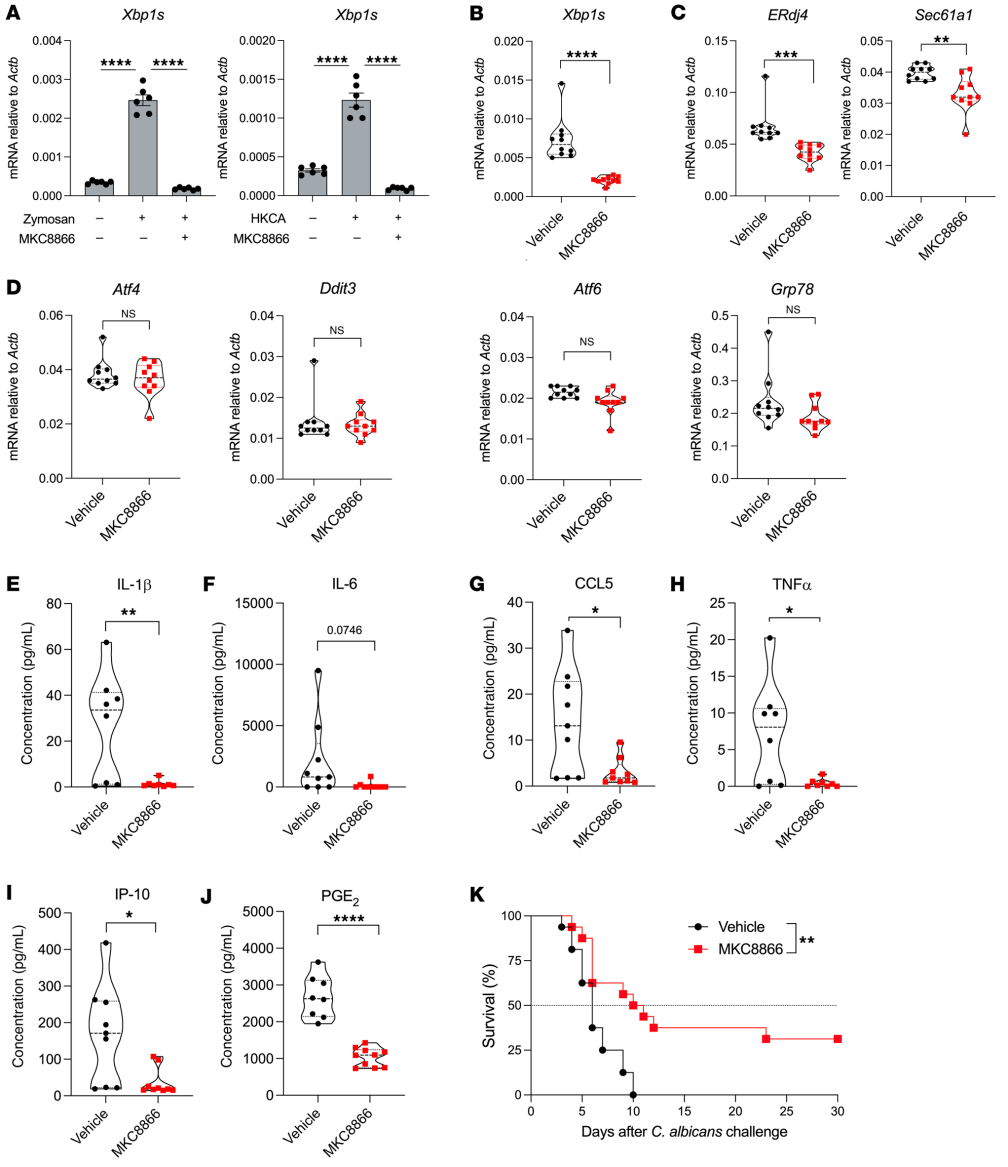
Pharmacological IRE1α inhibition controls kidney inflammation and extends survival in mice with systemic candidiasis. (**A**) Bone marrow–resident neutrophils from WT C57BL/6J mice (*n* = 6) were isolated and pretreated for 1 hour with vehicle control or MKC8866 (2.5 μM), followed by stimulation with either zymosan (25 μg/mL) or HKCA (MOI = 10) for 6 hours. *Xbp1s* transcript levels were determined by RT-qPCR and normalized to endogenous *Actb* expression in each sample. (**B**–**D**) WT C57BL/6J mice (*n* = 8–10 per group) were infected i.v. via tail vein injection with 10^5^ *C*. *albicans* cells. Twenty-four hours later, mice were treated once daily with vehicle control or MKC8866 (300 mg/kg) via oral gavage until postinfection day 5, and kidneys were resected 24 hours after the last treatment. Expression of the indicated transcripts was determined by RT-qPCR, and data were normalized to endogenous *Actb* expression in each sample. (**E**–**J**) Levels of the indicated proinflammatory factors were evaluated in the same kidney samples used in **B**–**D**. (**K**) WT C57BL/6J mice (*n* = 16 per group) were infected via tail vein injection with 10^5^ *C*. *albicans* cells. After 24 hours, mice were treated once daily with vehicle control or MKC8866 (300 mg/kg) via oral gavage for up to 10 days, and host survival was monitored thereafter. Data are shown as the mean ± SEM. *P < 0.05, **P < 0.005, ***P < 0.0005, and ****P < 0.0001, by 1-way ANOVA with Tukey’s test (**A**), 2-tailed Student’s *t* test (**B**–**J**), and log-rank test (**K**).

We next tested whether targeting IRE1α pharmacologically could control kidney inflammation and disease progression in mice systemically infected with *C*. *albicans*. To this end we used MKC8866, a selective small-molecule inhibitor of IRE1α that has been shown to control the detrimental hyperactivation of this ER stress sensor in cancer (66–68) and pain (8). Importantly, MKC8866 specifically targets the RNase domain of mammalian IRE1α and does not affect the function of RNase A or RNase L (68). In vitro treatment with MKC8866 inhibited IRE1α activation in primary neutrophils stimulated with zymosan or HKCA (Figure 7A). To test the efficacy of this compound in vivo, WT C57BL/6J mice were challenged i.v. with 10^5^ *C*. *albicans* SC5314 cells and 24 hours later, the mice were treated daily via oral gavage with vehicle control or MKC8866 for 5 consecutive days. Whole kidney tissue was analyzed 16 hours after the last dose. MKC8866 administration significantly reduced the levels of *Xbp1s* in the kidneys of *C*. *albicans*–infected mice (Figure 7B). Accordingly, the expression of IRE1α/XBP1-dependent target genes in the UPR, such as *ERdj4* and *Sec61a1,* was also significantly decreased in the kidneys from treated mice (Figure 7C), whereas the expression of UPR genes controlled by other ER stress sensors remained unaltered (Figure 7D). Consistent with our findings using IRE1α-deficient mice (Figures 3 and 6), therapeutic MKC8866 administration mitigated the production of IL-1β, IL-6, CCL5, TNF-α, IP-10, and PGE_2_ in the kidneys of infected mice (Figure 7, E–J) and extended their overall survival, with approximately 30% of treated hosts cured of the disease (Figure 7K). Hence, controlling IRE1α overactivation using a selective small-molecule inhibitor restricted the immunopathogenic progression of invasive candidiasis in mice.

## Discussion

**Figure 8:**
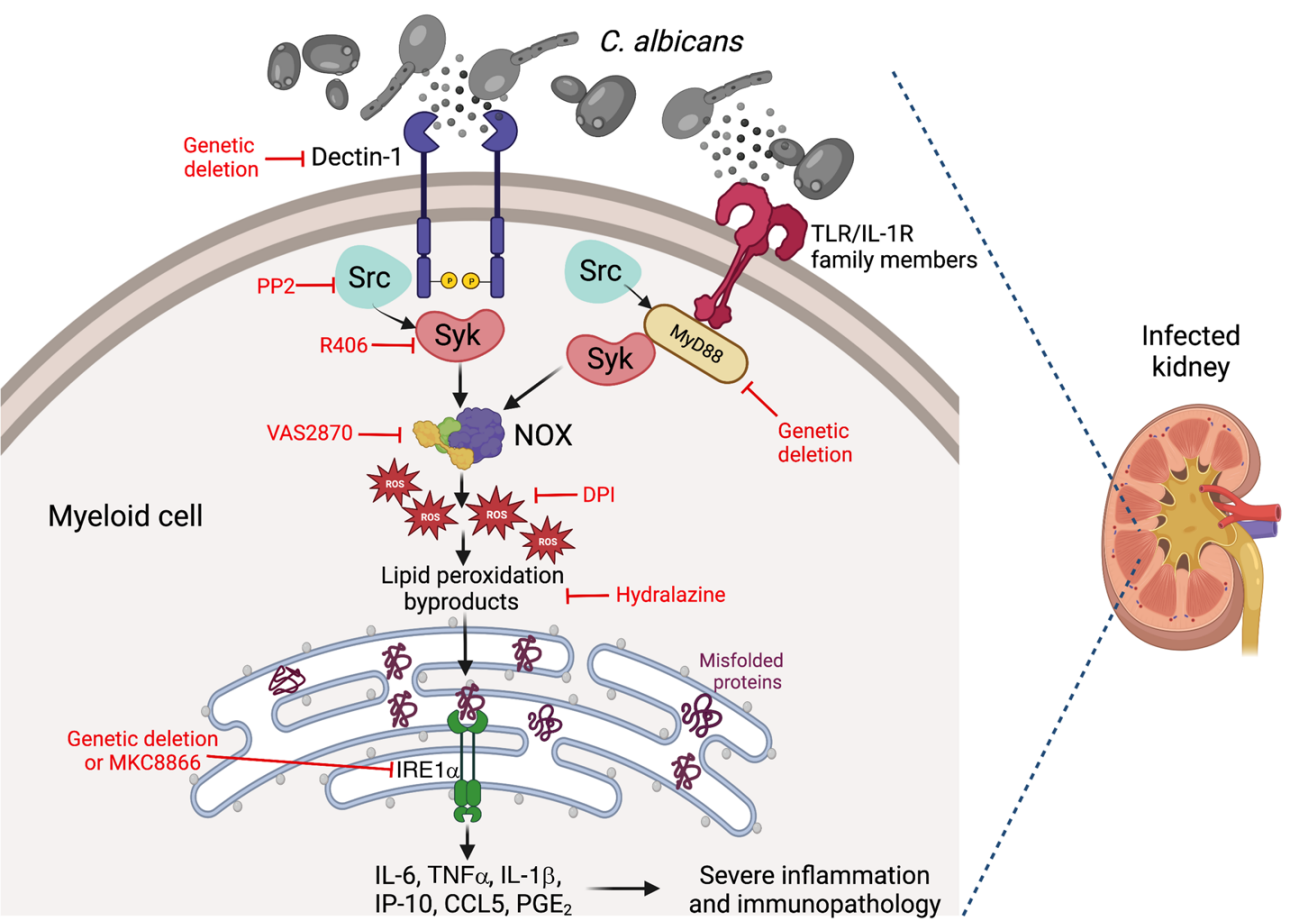
Proposed model. *C*. *albicans* activates parallel dectin-1 and TLR/IL-1R–MyD88 signaling in neutrophils and monocytes, triggering potent Src-Syk-NOX activation. Abundant ROS produced by these pathways engender lipid peroxidation byproducts that provoke ER stress and fuel IRE1α overactivation. Enhanced IRE1α signaling in kidney-resident myeloid cells of mice with systemic candidiasis mediates the overexpression of multiple inflammatory factors that promote kidney tissue damage and lethal disease progression.

The present study uncovers a detrimental role for innate IRE1α overactivation in systemic candidiasis. We determined that sensing of *C*. *albicans* by dectin-1 and the TLR/IL-1R adaptor protein MyD88 in myeloid cells triggered inflammatory IRE1α activation via ROS generated by the Src-Syk-NOX pathway (Figure 8, proposed model). The central role of Syk in this process is consistent with its involvement in 2 additional processes that directly impinge on ROS production: the release of arachidonate, which activates NOX by acting at multiple steps (69), and the generation of the NOX substrate NADPH via the citrate-pyruvate shuttle (70, 71). Accordingly, blocking ROS generation by NOX, or sequestering lipid peroxidation byproducts such as 4-HNE, abolished IRE1α activation in neutrophils and monocytes exposed to *C*. *albicans*. Ferroptosis, a type of cell death that implicates lipid peroxidation and the generation of 4-HNE, was recently shown to drive immunopathology during candidiasis (72). Hence, additional research is necessary to evaluate a potential crosstalk between ferroptosis and UPR overactivation in the immunopathogenesis of systemic candidiasis.

Our study focused on the role of immune-intrinsic IRE1α in candidemia based on results generated using ERAI reporter mice, which highlighted preferential overactivation of this ER stress sensor in kidney-infiltrating DCs, monocytes, and neutrophils during the early stages of invasive candidiasis. Yet, the innate response to this renal infection is a coordinated process involving both immune cells and kidney-resident cells, such as renal tubular epithelial cells (30, 73). Thus, it is possible that basal IRE1α activity in nonhematopoietic cells present in the kidney may also contribute to the observed pathological effects, a possibility that warrants further investigation.

Elder and colleagues reported that β-glucans can activate dectin-1, Syk, NF-κB, and p38 signaling, as well as the IRE1α and PERK arms of the UPR in human monocyte–derived DCs, to sustain the expression of thymic stromal lymphopoietin (74). Additional studies documented UPR activation by β-glucans, which promoted IL-23 production in macrophages and DCs (14, 15, 75). Nonetheless, the function of leukocyte-intrinsic IRE1α signaling in systemic candidiasis had not, to our knowledge, been established.

Kidney immunopathology in candidiasis involves a complex interplay of immune responses and fungal virulence factors. The presence of *C*. *albicans* in the kidney can trigger a potent inflammatory response characterized by the infiltration of innate immune cells that release cytokines, chemokines, ROS, and proteases, which can damage surrounding tissues (30). This process can lead to the recruitment of additional immune cells to the site of infection and promote tissue damage, leading to acute kidney injury and even kidney failure (30). Previous studies identified that host factors, such as the suppressor of TCR signaling 1 and 2 (Sts1/-2) (33), ubiquitin ligase Casitas C lymphoma-b (CBLB) (76, 77), and c-Jun N-terminal kinase-1 (JNK-1) (78), promote immunopathology upon systemic *C*. *albicans* infection. In addition, global deletion of the chemokine receptor 1 (CCR1) or IFNAR-1 was shown to reduce immunopathology without affecting fungal burden. In this context, CCR1 regulates the recruitment of neutrophils to the kidney in the late phase of systemic *C*. *albicans* infection (35), while IFNAR-1 dictates the recruitment of Ly6C^hi^ inflammatory monocytes to infected kidneys by driving the expression of CCL2 and KC (also known as CXCL1) (34).

Our study now indicates that IRE1α activation sculpted transcriptional programs in kidney-infiltrating neutrophils and monocytes that enable the simultaneous overexpression of major inflammatory factors, such IL-1β, IL-1α, IL-6, TNF-α, PGE_2_, CCL5, IP-10/CXCL10, and MCP-1/CCL2, which contribute to the renal immunopathology observed in systemic candidiasis (28, 34, 79). Indeed, we found that the levels of these mediators were decreased in the kidneys of IRE1α conditional-KO hosts infected with *C*. *albicans*. These effects were associated with reduced kidney immunopathology at later stages of infection and increased overall survival, leading to a full recovery in approximately 30% of challenged hosts. Thus, our study unveils the ER stress sensor IRE1α as a major immunopathogenic driver of fatal candidemia (Figure 8).

We also determined that controlling IRE1α pharmacologically using MKC8866 attenuated the overexpression of inflammatory factors in the kidney and extended the overall survival of mice with systemic candidiasis, thus phenocopying the effects of conditional IRE1α deficiency in leukocytes. These results are noteworthy, given that a similar inhibitor of the IRE1α RNase domain, named ORIN1001, is currently undergoing clinical trials in patients with cancer (ClinicalTrials.gov identifier: NCT03950570) and pulmonary fibrosis (ClinicalTrials.gov identifier: NCT04643769). Therefore, we propose that targeting IRE1α pharmacologically represents a major opportunity for controlling the detrimental hyperinflammatory condition provoked by systemic *C*. *albicans* infection. In patients, invasive candidiasis causes high mortality despite the availability of antifungal drugs (80, 81). Attenuating IRE1α signaling to mitigate overt inflammation and kidney tissue damage, while preserving the antifungal activity of immune cells, may therefore be useful to manage systemic candidiasis more effectively in the clinic. The role of IRE1α in adaptive immune responses to *C*. *albicans* in mucosal, dermal, or vaginal infections (82) remains elusive and warrants further investigation.

## Methods

### Transgenic mice.

C57BL/6J, *Vav1^Cre^*, *Fcer1g^–/–^*, *Atf6^fl/fl^*, *Myd88^−/−^*, and *Eif2ak3^fl/fl^* mice were obtained from The Jackson Laboratory. ERAI and *Ern1^fl/fl^* mice have been previously described by our group (8, 38, 83). We generated conditional-KO mice lacking ATF6, PERK, or IRE1α in leukocytes by crossing *Atf6^fl/fl^*, *Eif2ak3^fl/fl^*, or *Ern1^fl/fl^* mice, respectively, with the *Vav1^Cre^* strain that allows selective gene deletion in hematopoietic cells (8, 62). To generate conditional-KO mice lacking IRE1α specifically in neutrophils, *Ern1^fl/fl^* mice were crossed with the *Mrp8^Cre^* strain (64). *Clec7a^–/–^* mice were provided by Y. Iwakura (84). *Clec7a^–/–^ Fcer1g^–/–^* double-KO mice were generated by crossing *Fcer1g^–/–^* mice with *Clec7a^–/–^* mice. All strains used in this study were on the C57BL/6 genetic background. Male and female mice were used at 8–12 weeks of age for all experiments. Mice were maintained in ventilated cages under specific pathogen–free conditions at the animal facilities of Memorial Sloan Kettering Cancer Center and Weill Cornell Medical College.

### Isolation of neutrophils and monocytes from mouse bone marrow.

Untouched neutrophils or monocytes were isolated from the bone marrow of WT or transgenic mice by negative selection using the Neutrophil Isolation Kit (Miltenyi Biotec, catalog 130-097-658) or the Monocyte Isolation kit (Miltenyi Biotec, catalog 130-100-629), respectively. Briefly, the bone marrow was flushed in complete RPMI media (RPMI + l-glutamine + 10% FBS + HEPES + sodium pyruvate + nonessential amino acids + β mercaptoethanol + penicillin/streptomycin). Erythrocytes were depleted using ACK lysis buffer, and cells were then resuspended in MACS buffer (0.5% BSA, 1× PBS, 2 mM EDTA). Primary cell isolation was carried out following Miltenyi Biotec’s protocols. In all cases, purity was confirmed to be greater than 90% by FACS analysis.

### RNA isolation, RT-qPCR, and Xbp1 splicing assays.

Total RNA was isolated using the RNeasy Mini kit or QIAzol lysis reagent (QIAGEN). Total RNA (0.1–1 μg) was reverse transcribed to generate cDNA using the qScript cDNA Synthesis kit (Quantabio). RT-qPCR was performed using the PerfeCTa SYBR green fastmix (Quantabio) on a QuantStudio 6 Flex real-time PCR instrument (Applied Biosystems). Normalized gene expression was calculated by the comparative threshold cycle method using *Actb* as the endogenous housekeeping gene control. *Xbp1* splicing assays were performed as previously described (8, 85). PCR products were separated by electrophoresis through a 2.5% agarose gel and visualized by GelRed staining. All primers used in this study are described in Supplemental Table 2.

### C. albicans and β-glucans.

We used the *C*. *albicans* clinical isolate SC5314 (86), which was grown in yeast peptone dextrose (YPD) broth (Amresco) for 16 hours at 30°C. HKCA was prepared by heating yeast cultures at 70°C for 30 minutes. Hot alkali-treated zymosan (HATZ) and curdlan were purchased from Invivogen (catalog tlrl-zyd and tlrl-curd, respectively). Zymosan was obtained from MilliporeSigma (catalog Z4250-1G). For in vitro treatments, neutrophils were stimulated with the agonists described above or cocultured with live *C*. *albicans* or HKCA in 96-well plates at 37°C for the indicated durations and MOIs.

### ROS analysis and targeting.

Intracellular ROS measurements were performed by labeling with 10 μM DCFDA (Thermo Fisher Scientific, catalog C6827). To define the source of ROS production, 250,000 bone marrow–resident neutrophils were pretreated for 30 minutes at 37°C with 10 μM VAS-2870 (Enzo Biosciences, catalog BML-E1395-001) or 10 μM DPI (MilliporeSigma, catalog D2926-10MG). Cells were then loaded with DCFDA for 30 minutes at 37°C and subsequently treated with HKCA, zymosan, or PMA at the indicated MOI or concentrations. 

To determine the role of mitochondrial ROS and lipid peroxidation byproducts, bone marrow neutrophils were pretreated with Mito-TEMPO (10 μM, MilliporeSigma, catalog SML0737-5MG), MitoQ (2 μM, Cayman Chemicals, catalog 89950-1MG), or hydralazine (100 μg/mL, MilliporeSigma, catalog H1753-5G) for 30 minutes and then stimulated with HKCA or zymosan for the indicated durations. After treatment, cells were processed for RNA extraction or flow cytometric analysis.

### ThT staining.

Bone marrow–derived neutrophils were plated in a 96-well plate at a density of 250,000 cells per well. The cells were then stimulated with HKCA (MOI = 1, 3, or 5), zymosan (5, 10, or 25 μg/mL), or left untreated for 6 hours at 37°C. After treatment, the supernatant was removed by centrifuging the plate at 500*g* for 5 minutes. To measure intracellular misfolded protein aggregates, cells were labeled with the cell-permeable fluorescent dye ThT (Abcam, catalog ab120751; stock 50 mM in DMSO, final concentration 5 μM in PBS). The cells were resuspended in 200 μL ThT staining solution and incubated for 15 minutes at room temperature in the dark. Cells were then washed twice with FACS buffer. Subsequently, the cells were Fc-γ receptor blocked using TruStain FcX (BioLegend, anti–mouse CD16/32, clone 93, catalog 101319) for 10 minutes at 4°C, and then stained for surface markers at 4°C in the dark for 30 minutes with the following antibodies: anti-CD45 (clone 30-F11 [RUO], BD Biosciences, catalog 562420), anti-CD11b (clone M1/70, BioLegend, catalog 101228), and anti-Ly6G (1A8 [RUO], BD Biosciences, catalog 741813). Cells were then washed with FACS buffer and stained with LIVE/DEAD Fixable Near-IR Dead Cell Stain (Thermo Fisher Scientific, catalog L34975) for live/dead discrimination. Flow cytometry was performed using a Fortessa-X20 instrument (BD Biosciences).

### Assessment of neutrophil effector functions.

Phagocytosis assays were performed by exposing primary neutrophils to FITC-labeled zymosan (Thermo Fisher Scientific, catalog Z2841) at a 1:1 ratio for 30 minutes at 37°C, followed by a double wash with FACS buffer. Cells were then analyzed on an LSR II flow cytometer (BD Biosciences). ROS generation was quantified using DCFDA, as described above. Briefly, 250,000 bone marrow neutrophils from mice of the indicated genotypes were labeled with 10 μM DCDA for 30 minutes at 37°C. Cells were then stimulated with either HKCA (MOI = 5), zymosan (25 μg/mL), or PMA (50 nM, as a positive control) for 1 hour at 37°C. Samples were subsequently analyzed on an LSR II flow cytometer. To determine MPO production, bone marrow neutrophils were isolated from mice of the indicated genotype and stimulated with either HKCA (MOI = 5) or zymosan (25 μg/mL) for 6 hours in 96-well plates at 37°C. Supernatants were collected after treatment and stored at –80°C until analyzed. Supernatants (25 μL) were used to measure MPO using the MPO Mouse ELISA Kit (Thermo Fisher Scientific, catalog EMMPO). Plates were read at 450 nm using a Varioskan Instrument.

DNA release was measured using Sytox Green cell-impermeable nucleic acid stain (Thermo Fisher Scientific, catalog S7020) as described before (87, 88). Bone marrow neutrophils were labeled with 5 μM Sytox Green and then seeded in black 96-well plates at 100,000 cells/well. Cells were subsequently stimulated with HKCA (MOI = 20) or zymosan (25 μg/mL) for 8 hours. Treatment with PMA (100 nM) was used as a positive control. The fluorescence generated was measured using a Varioskan instrument (Thermo Fisher Scientific).

Killing assays were performed as described previously (79). Briefly, primary neutrophils were mixed 1:1 with *C*. *albicans* yeast cells under gentle agitation at 37°C. Aliquots of 10 mL were taken at different time points, lysed by resuspension in water, and fungal CFUs were then determined by serial dilutions on YPD agar plates. The killing percentage was evaluated by calculating the number of surviving *C*. *albicans* in neutrophil cocultures divided by total *C*. *albicans* without neutrophils.

### In vivo C. albicans infection.

*C*. *albicans* SC5314 was cultured as described above and washed 3 times with 1× PBS. Yeast cells were then counted on a hemocytometer and suspended at a concentration of 5 × 10^5^ cells/mL in PBS. WT or transgenic mice of the indicated genotypes were i.v. challenged with 100,000 *C*. *albicans* SC5314 cells in 200 μL PBS via tail vein injection. Mice were humanely euthanized at different time points after challenge for downstream analyses or monitored daily to determine overall survival rates.

### Assessment of fungal burden.

Mice were humanely euthanized, and kidneys were aseptically removed at the indicated time points following *C*. *albicans* infection. Kidneys were weighed and ground in 1× PBS with a pestle in 10 cm plates. Homogenates were collected, serially diluted, and plated on YPD agar plates (2% agar) containing chloramphenicol (34 mg/mL) and gentamycin (34 mg/mL). CFU were determined 48 hours after incubation at 30°C. Fungal burden was calculated as CFU/gram of kidney tissue.

### Analysis of immune cells in kidney, blood, spleen, and bone marrow.

Kidney single-cell suspensions were obtained as previously reported (35) with some modifications. Briefly, mice were euthanized before or at different time points after infection, and kidneys were excised and collected in petri dishes containing 5 mL RPMI plus DNase I (0.05 mg/mL, MilliporeSigma). The kidney was minced on a cell dissociation mesh (Bellcoglass, SKU: 1985-00100) to obtain a single-cell suspension. The suspension was then passed through a 70 mm cell strainer and washed with 20 mL cold RPMI containing 10% FBS. This cell suspension was centrifuged at 500*g* for 8 minutes at 4°C. The supernatant was discarded, and RBCs were lysed using 5 mL ACK lysis buffer for 3 minutes, followed by addition of 20 mL 1× cold PBS. Cell suspensions were immediately passed through a 40 mm cell strainer and again centrifuged at 500*g* for 8 minutes at 4°C. Supernatants were discarded, and cell pellets were resuspended in 8 mL 40% Percoll. This cell suspension was then slowly and carefully overlaid onto 3 mL 70% Percoll in a 15 mL Falcon tube. The gradient was centrifuged at 2,000 rpm for 30 minutes at room temperature without brakes. The enriched leukocyte fraction was collected from the interphase and washed twice with FACS buffer (1× PBS, 2% FBS and 2 mM EDTA) at 500*g* for 8 minutes at 4°C. Spleen single-cell suspensions were obtained by directly grinding the spleen onto a 70 mm cell strainer. Bone marrow cells were obtained by flushing the tibiae and femurs of mice with 1× PBS. Circulating leukocytes were analyzed by collecting blood by cardiac puncture. In all cases, RBCs were depleted by treatment with ACK lysis buffer.

Analysis of leukocyte populations in kidney, spleen, blood, and bone marrow single-cell suspensions was performed by flow cytometry using fluorochrome-conjugated antibodies purchased from BioLegend, unless stated otherwise. Cells were Fc-γ receptor blocked using TruStain FcX (anti–mouse CD16/32, clone 93, catalog 101319) and then stained for surface markers at 4°C in the dark for 30 minutes with the following antibodies: anti-CD45 (clone 30-F11 [RUO], BD Biosciences, catalog 562420), anti-CD3 (clone 17A2, catalog 100216), anti-CD19 (clone ID3, BD Biosciences, catalog 612781), anti-CD11b (clone M1/70, catalog 101257), anti-F4/80 (clone BM8, catalog 123110), anti-CD11c (clone N418, BD Biosciences, catalog 744180), anti–I-A/I-E (clone M5/114, catalog 107620), anti-Ly6c (clone HK1.4, catalog 128041), anti-Ly6G (clone 1A8, Tonbo Biosciences, catalog 20-1276-U100), and anti-NK1.1 (clone PK136, catalog 108748). Cells were then washed with 1× PBS and stained with DAPI for live/dead discrimination. Flow cytometry was performed using a Fortessa-X20 instrument (BD Biosciences), and data were analyzed using FlowJo software (TreeStar).

### RNA-Seq and bioinformatics analyses.

Kidney-infiltrating neutrophils and monocytes were sorted from *Ern1^fl/fl^* or *Ern1^fl/fl^* *Vav1^Cre^* mice 36 hours after i.v. challenge with *C*. *albicans* SC5314. Neutrophils (CD45^+^Ly6g^hi^Ly6c^lo^CD11b^+^CD3^–^CD19^–^F4/80^–^CD11c^–^) and monocytes (CD45^+^Ly6c^hi^CD11b^+^Ly6g^–^CD3^–^CD19^–^F4/80^–^CD11c^–^) were sorted on a FACSAria Instrument (BD Biosciences) and processed for RNA isolation using the RNeasy Plus Micro Kit (QIAGEN). All samples passed quality controls as assessed by the Agilent Bioanalyzer 2100, and mRNA libraries were generated and sequenced at the Weill Cornell Genomics Resources Core Facility. Raw sequenced reads were pseudoaligned to the mouse reference genome (UCSC mm10) using Kallisto (89). Then, transcript abundance was quantified to attain raw counts. Raw counts obtained from Kallisto were used to identify DEGs using DESeq2 R package (90). Genes with very low expression values (base mean ≤15) were filtered out. Next, genes that satisfied the following 2 parameters were considered to be DEGs: (a) genes with an adjusted *P* value of less than 0.05 and (b) a |log_2_ fold change| of greater than 1.0. Volcano plots were generated to visually display the most significantly DEGs using statistical significance versus fold change. To reveal the molecular function of DEGs, Hallmark gene sets from the MSigDB were used (91, 92). The analyses were carried out on the Broad Institute’s Gene Set Enrichment Analysis (GSEA) public server (http://www.gsea-msigdb.org/gsea/) (93). Single-sample GSEA (ssGSEA) was used to verify whether a given pathway was coordinately up- or downregulated in a cohort. ssGSEA computes an enrichment score for each gene set, and the score denotes the activity level of a biological process. The analysis was carried out on the Broad Institute’s GenePattern public server (https://www.genepattern.org/modules/docs/ssGSEAProjection/4). A Pan-UPR gene set (*Hyou1*, *Hspa14*, *Sec61a1*, *Sec24d*, *Hspa13*, *Sec24c*, *Ambra1*, *Surf4*, *Atg13*, *Xbp1, P4hb*, *Fam129a*, *Pdia4*, *Spcs3*, *Surf6*, *Atf6*, *Dapk1*, *Dnajb9*, *Dnajc3*, *Mfn2*, *Pdia6*, *Ccdc47*, *Dnajc14*, *Sil1*, *Sec16a*, *Tmx3*, *Sec23b*, *Sec31a*, *Gosr2*, *Asns*, *Atf4*, *Ddit3*, *Ddit4*, *Edem1*) and a RIDD gene set (*Blosc1s1*, *Hgsnat*, *Pdgfna*, *Tapbp*, *Galnt2*, *Ergic3*, *Lamp*, *Tpp1*, *Txndc5*, *Erp44*, *Rpn1*, *Idi1*, *Slc35f5*, *Pmp22*, *Col6a1*, *Pdgfr*) were used for the analyses. (Gene ratio = the number of genes from input that are included in a given gene set/the total number of genes in a given gene set.) For all RNA-Seq analyses, statistical significance was set at a 2-tailed *P* value of less than 0.05, and the analyses were conducted using R software (R Foundation for Statistical Computing).

### Histopathology.

Following euthanasia, the kidneys were immediately removed and harvested. For each mouse, half of 1 kidney was collected and immediately fixed in 10% neutral-buffered formalin. Then, the samples were transferred to 70% ethanol, routinely processed in alcohol and xylene, sectioned at 4 μm thickness, and stained with H&E. Immunohistochemical analyses for MPO (Dako, A0398, 1:1,000, following heat-induced epitope retrieval [HIER] in a pH 6.0 buffer) and CD45 (BD Pharmigen 550539, 1:250, HIER pH 6.0) were performed on a Leica Bond RX automated stainer using the Bond Polymer Refine detection system (Leica Biosystems, DS9800). The chromogen was DAB, and sections were counterstained with hematoxylin.

Specimens were histologically assessed under light microscopy for the detection of lesions. All sections were scored according to the method reported by Wirnsberger et al. with modifications (77). Specifically, the proportion of renal parenchyma affected by inflammatory changes (tubulointerstitial nephritis and/or pyelonephritis), along with other tubular or interstitial alterations, were recorded and scored as not significant (score = 0), less than 10% renal parenchyma affected (score = 1), 10%–25% renal parenchyma affected (score = 2), 26%–50% renal parenchyma affected (score = 3), and greater than 50% renal parenchyma affected (score = 4).

### Analysis of inflammatory factors in kidney samples.

Cytokine and chemokine levels in kidneys of mice with systemic *C*. *albicans* infection were measured by digesting the excised organ in 1 mL LiberaseTL and DNAse I solution at 37°C for 20 minutes, followed by straining through a 40 mm filter, and centrifugation at 12,000*g* for 45 minutes at 4°C. Supernatants were collected and stored at –80°C. Cytokine/chemokine analysis of these samples was performed at EVE Technologies using the Mouse Cytokine Array/Chemokine Array 44-Plex (MD44) immunoassay. PGE_2_ levels in kidney supernatants were measured using the PGE_2_ ELISA kit (Enzo Lifesciences, catalog ADI-900-001). Plates for PGE_2_ were read at 405 nm using a Varioskan instrument (Thermo Fisher Scientific), as we previously reported (8).

### In vivo treatment with MKC8866.

C57BL/6J mice were challenged i.v. with 100,000 *C*. *albicans* SC5314 yeast cells in 200 μL PBS via tail vein injection. After 24 hours, infected mice were treated daily via oral gavage with 300 mg/kg MKC8866 (custom manufactured at WuXi AppTec or purchased from MedChemExpress, catalog HY-104040) for up to 10 days. The vehicle used for MKC8866 administration was 1% microcrystalline cellulose in 50% sucrose solution, which was prepared by heating at 60°C and stirring until complete solubilization. MKC8866 was added to this vehicle to obtain a final concentration of 30 mg/mL, and the mix was sonicated for 1 hour at 42 kHz at room temperature to generate a homogeneous suspension. The volume of MKC8866 was given such that each mouse received a dose of 300 mg/kg body weight.

### Statistics.

All statistical analyses were performed using GraphPad Prism 8 (GraphPad Software). Comparison between 2 groups were assessed using an unpaired or paired (for matched comparisons) 2-tailed Student’s *t* test. The Mann-Whitney *U* test was used to analyze histopathological scoring. Multiple comparisons were evaluated by 1-way ANOVA, including Tukey’s or Dunnett’s multiple-comparison tests. Where applicable, data represent the mean ± SEM. Host survival after *C*. *albicans* infection was analyzed using the log-rank (Mantel-Cox) test. *P* values of less than 0.05 were considered statistically significant.

### Study approval.

All in vivo experimentation in mice complied with the Weill Cornell IACUC under an approved protocol (protocol no. 2016-0052).

### Supplemental data.

Values for all data points found in the figures can be found in the Supplemental Supporting Data Values file.

## Author contributions

DA and SC designed and performed the research, analyzed data, and wrote the manuscript. BAC analyzed and interpreted the RNA-Seq data. AE, TAS, SMH, CSC, CS, CT, and LVU performed in vitro and in vivo experiments. JJFR analyzed data. SFS performed all histopathological analyses and interpreted data. TI provided ERAI and Ern1-floxed transgenic mice. EARS, MSC, DKM, and IDI provided experimental tools, models, and analyzed the data. TMH and JRCR conceived and designed the research, analyzed data, and wrote the manuscript.

## Supplementary Material

Supplemental data

Supporting data values

## Version 1 07/11/2023

In-Press Preview

## Version 2 09/01/2023

Electronic publication

## References

[1] Hetz C (2020). Mechanisms, regulation and functions of the unfolded protein response. Nat Rev Mol Cell Biol.

[2] Celli J, Tsolis RM (2015). Bacteria, the endoplasmic reticulum and the unfolded protein response: friends or foes?. Nat Rev Microbiol.

[3] Bettigole SE, Glimcher LH (2015). Endoplasmic reticulum stress in immunity. Annu Rev Immunol.

[4] Chen X, Cubillos-Ruiz JR (2021). Endoplasmic reticulum stress signals in the tumour and its microenvironment. Nat Rev Cancer.

[5] Hetz C (2019). Pharmacological targeting of the unfolded protein response for disease intervention. Nat Chem Biol.

[6] Di Conza G Control of immune cell function by the unfolded protein response. Nat Rev Immunol.

[7] Choi JA, Song CH (2019). Insights into the role of endoplasmic reticulum stress in infectious diseases. Front Immunol.

[8] Chopra S (2019). IRE1α-XBP1 signaling in leukocytes controls prostaglandin biosynthesis and pain. Science.

[9] Casadevall A, Pirofski LA (2000). Host-pathogen interactions: basic concepts of microbial commensalism, colonization, infection, and disease. Infect Immun.

[10] Jabra-Rizk MA (2016). Candida albicans pathogenesis: fitting within the host-microbe damage response framework. Infect Immun.

[11] Qiu Q (2013). Toll-like receptor-mediated IRE1α activation as a therapeutic target for inflammatory arthritis. EMBO J.

[12] Martinon F (2010). TLR activation of the transcription factor XBP1 regulates innate immune responses in macrophages. Nat Immunol.

[13] Rosen DA (2019). Modulation of the sigma-1 receptor-IRE1 pathway is beneficial in preclinical models of inflammation and sepsis. Sci Transl Med.

[14] Marquez S (2017). Endoplasmic reticulum stress sensor IRE1α Enhances IL-23 expression by human dendritic cells. Front Immunol.

[15] Mogilenko DA (2019). Metabolic and innate immune cues merge into a specific inflammatory response via the UPR. Cell.

[16] Marakalala MJ (2011). Dectin-1: a role in antifungal defense and consequences of genetic polymorphisms in humans. Mamm Genome.

[17] Verma A (2014). Adaptive immunity to fungi. Cold Spring Harb Perspect Med.

[18] Drummond RA, Brown GD (2011). The role of Dectin-1 in the host defence against fungal infections. Curr Opin Microbiol.

[19] Yang H (2011). CARD9 Syk-dependent and Raf-1 Syk-independent signaling pathways in target recognition of Candida albicans by Dectin-1. Eur J Clin Microbiol Infect Dis.

[20] Graham DB (2007). Neutrophil-mediated oxidative burst and host defense are controlled by a Vav-PLCgamma2 signaling axis in mice. J Clin Invest.

[21] Segal BH (2012). Regulation of innate immunity by NADPH oxidase. Free Radic Biol Med.

[22] Mocsai A (2002). Syk is required for integrin signaling in neutrophils. Immunity.

[23] Saijo S, Iwakura Y (2011). Dectin-1 and Dectin-2 in innate immunity against fungi. Int Immunol.

[24] Nguyen GT (2017). Neutrophils to the ROScue: mechanisms of NADPH oxidase activation and bacterial resistance. Front Cell Infect Microbiol.

[25] Netea MG (2015). Immune defence against Candida fungal infections. Nat Rev Immunol.

[26] Reid DM (2009). Pattern recognition: recent insights from Dectin-1. Curr Opin Immunol.

[27] Mayer FL (2013). Candida albicans pathogenicity mechanisms. Virulence.

[28] MacCallum DM (2009). Massive induction of innate immune response to Candida albicans in the kidney in a murine intravenous challenge model. FEMS Yeast Res.

[29] Parker JC (1976). , et al. Pathobiologic features of human candidiasis. A common deep mycosis of the brain, heart and kidney in the altered host. Am J Clin Pathol.

[30] Jawale CV, Biswas PS (2021). Local antifungal immunity in the kidney in disseminated candidiasis. Curr Opin Microbiol.

[31] Lionakis MS (2011). Organ-specific innate immune responses in a mouse model of invasive candidiasis. J Innate Immun.

[32] Spellberg B (2005). Mice with disseminated candidiasis die of progressive sepsis. J Infect Dis.

[33] Naseem S (2015). Protection from systemic Candida albicans infection by inactivation of the Sts phosphatases. Infect Immun.

[34] Majer O (2012). Type I interferons promote fatal immunopathology by regulating inflammatory monocytes and neutrophils during Candida infections. PLoS Pathog.

[35] Lionakis MS (2012). Chemokine receptor Ccr1 drives neutrophil-mediated kidney immunopathology and mortality in invasive candidiasis. PLoS Pathog.

[36] Desai JV, Lionakis MS (2018). The role of neutrophils in host defense against invasive fungal infections. Curr Clin Microbiol Rep.

[37] Ngo LY (2014). Inflammatory monocytes mediate early and organ-specific innate defense during systemic candidiasis. J Infect Dis.

[38] Iwawaki T (2004). A transgenic mouse model for monitoring endoplasmic reticulum stress. Nat Med.

[39] Beriault DR, Werstuck GH (2013). Detection and quantification of endoplasmic reticulum stress in living cells using the fluorescent compound, Thioflavin T. Biochim Biophys Acta.

[40] Sicari D (2020). A guide to assessing endoplasmic reticulum homeostasis and stress in mammalian systems. FEBS J.

[41] Lee AH (2003). XBP-1 regulates a subset of endoplasmic reticulum resident chaperone genes in the unfolded protein response. Mol Cell Biol.

[42] Brown GD (2006). Dectin-1: a signalling non-TLR pattern-recognition receptor. Nat Rev Immunol.

[43] Goodridge HS (2009). Beta-glucan recognition by the innate immune system. Immunol Rev.

[44] Brown GD (2003). Dectin-1 mediates the biological effects of beta-glucans. J Exp Med.

[45] Tang J (2018). Regulation of C-type lectin receptor-mediated antifungal immunity. Front Immunol.

[46] Gil ML, Gozalbo D (2009). Role of Toll-like receptors in systemic Candida albicans infections. Front Biosci (Landmark Ed).

[47] Netea MG (2004). Toll-like receptor 2 suppresses immunity against Candida albicans through induction of IL-10 and regulatory T cells. J Immunol.

[48] Yi YS (2021). Syk-MyD88 axis is a critical determinant of inflammatory-response in activated macrophages. Front Immunol.

[49] Brandvold KR (2012). Development of a highly selective c-Src kinase inhibitor. ACS Chem Biol.

[50] Hanke JH (1996). Discovery of a novel, potent, and Src family-selective tyrosine kinase inhibitor. Study of Lck- and FynT-dependent T cell activation. J Biol Chem.

[51] Braselmann S (2006). R406, an orally available spleen tyrosine kinase inhibitor blocks fc receptor signaling and reduces immune complex-mediated inflammation. J Pharmacol Exp Ther.

[52] Ruzza P (2009). Therapeutic prospect of Syk inhibitors. Expert Opin Ther Pat.

[53] Gross O (2009). Syk kinase signalling couples to the Nlrp3 inflammasome for anti-fungal host defence. Nature.

[54] Bae YS (2009). Macrophages generate reactive oxygen species in response to minimally oxidized low-density lipoprotein: toll-like receptor 4- and spleen tyrosine kinase-dependent activation of NADPH oxidase 2. Circ Res.

[55] Cubillos-Ruiz JR (2015). ER stress sensor XBP1 controls anti-tumor immunity by disrupting dendritic cell homeostasis. Cell.

[56] Vladykovskaya E (2012). Lipid peroxidation product 4-hydroxy-trans-2-nonenal causes endothelial activation by inducing endoplasmic reticulum stress. J Biol Chem.

[57] Kalyanaraman B (2012). Measuring reactive oxygen and nitrogen species with fluorescent probes: challenges and limitations. Free Radic Biol Med.

[58] Riganti C (2004). Diphenyleneiodonium inhibits the cell redox metabolism and induces oxidative stress. J Biol Chem.

[59] Wingler K (2012). VAS2870 is a pan-NADPH oxidase inhibitor. Cell Mol Life Sci.

[60] Hu Q (2018). The mitochondrially targeted antioxidant MitoQ protects the intestinal barrier by ameliorating mitochondrial DNA damage via the Nrf2/ARE signaling pathway. Cell Death Dis.

[61] To EE (2020). Mitochondrial reactive oxygen species contribute to pathological inflammation during influenza A virus infection in mice. Antioxid Redox Signal.

[62] De Boer J (2003). Transgenic mice with hematopoietic and lymphoid specific expression of Cre. Eur J Immunol.

[63] Bettigole SE (2015). The transcription factor XBP1 is selectively required for eosinophil differentiation. Nat Immunol.

[64] Abram CL (2014). Comparative analysis of the efficiency and specificity of myeloid-Cre deleting strains using ROSA-EYFP reporter mice. J Immunol Methods.

[65] MacCallum DM (2009). Early-expressed chemokines predict kidney immunopathology in experimental disseminated Candida albicans infections. PLoS One.

[66] Logue SE (2018). Inhibition of IRE1 RNase activity modulates the tumor cell secretome and enhances response to chemotherapy. Nat Commun.

[67] Sheng X (2019). IRE1α-XBP1s pathway promotes prostate cancer by activating c-MYC signaling. Nat Commun.

[68] Zhao N (2018). Pharmacological targeting of MYC-regulated IRE1/XBP1 pathway suppresses MYC-driven breast cancer. J Clin Invest.

[69] Matono R (2014). Arachidonic acid induces direct interaction of the p67(phox)-Rac complex with the phagocyte oxidase Nox2, leading to superoxide production. J Biol Chem.

[70] Seegren PV (2020). Mitochondrial Ca^2+^ signaling is an electrometabolic switch to fuel phagosome killing. Cell Rep.

[71] Mancebo C (2022). Fungal patterns induce cytokine expression through fluxes of metabolic intermediates that support glycolysis and oxidative phosphorylation. J Immunol.

[72] Millet N (2022). IL-23 signaling prevents ferroptosis-driven renal immunopathology during candidiasis. Nat Commun.

[73] Ramani K (2018). Unexpected kidney-restricted role for IL-17 receptor signaling in defense against systemic Candida albicans infection. JCI Insight.

[74] Elder MJ (2016). TSLP production by dendritic cells is modulated by IL-1β and components of the endoplasmic reticulum stress response. Eur J Immunol.

[75] Rodriguez M (2014). The unfolded protein response and the phosphorylations of activating transcription factor 2 in the trans-activation of il23a promoter produced by β-glucans. J Biol Chem.

[76] Xiao Y (2016). Targeting CBLB as a potential therapeutic approach for disseminated candidiasis. Nat Med.

[77] Wirnsberger G (2016). Inhibition of CBLB protects from lethal Candida albicans sepsis. Nat Med.

[78] Zhao X (2017). JNK1 negatively controls antifungal innate immunity by suppressing CD23 expression. Nat Med.

[79] Greenblatt MB (2010). Calcineurin regulates innate antifungal immunity in neutrophils. J Exp Med.

[80] Brown GD (2012). Hidden killers: human fungal infections. Sci Transl Med.

[81] Tsay SV (2020). Burden of Candidemia in the United States, 2017. Clin Infect Dis.

[82] Conti HR (2014). Animal models for candidiasis. Curr Protoc Immunol.

[83] Iwawaki T (2009). Function of IRE1 alpha in the placenta is essential for placental development and embryonic viability. Proc Natl Acad Sci U S A.

[84] Saijo S (2007). Dectin-1 is required for host defense against Pneumocystis carinii but not against Candida albicans. Nat Immunol.

[85] Iwakoshi NN (2003). Plasma cell differentiation and the unfolded protein response intersect at the transcription factor XBP-1. Nat Immunol.

[86] Gillum AM (1984). Isolation of the Candida albicans gene for orotidine-5’-phosphate decarboxylase by complementation of S. cerevisiae ura3 and E. coli pyrF mutations. Mol Gen Genet.

[87] Behnen M (2014). Immobilized immune complexes induce neutrophil extracellular trap release by human neutrophil granulocytes via FcγRIIIB and Mac-1. J Immunol.

[88] Awasthi D (2019). Glycolysis dependent lactate formation in neutrophils: a metabolic link between NOX-dependent and independent NETosis. Biochim Biophys Acta Mol Basis Dis.

[89] Bray NL (2016). Near-optimal probabilistic RNA-seq quantification. Nat Biotechnol.

[90] Love MI (2014). Moderated estimation of fold change and dispersion for RNA-seq data with DESeq2. Genome Biol.

[91] Liberzon A (2011). Molecular signatures database (MSigDB) 3.0. Bioinformatics.

[92] Liberzon A (2015). The molecular signatures database (MSigDB) hallmark gene set collection. Cell Syst.

[93] Subramanian A (2005). Gene set enrichment analysis: a knowledge-based approach for interpreting genome-wide expression profiles. Proc Natl Acad Sci U S A.

