# Supplementary material for: Inflammatory ER stress responses dictate the immunopathogenic progression of systemic candidiasis
Source: J Clin Invest. 2023 Sep 1;133(17):e167359. doi: 10.1172/JCI167359 (PMC10471176; doi:10.1172/JCI167359)
Supplement: Supplemental data [file jci-133-167359-s009.pdf]

Supplementary Figure 1

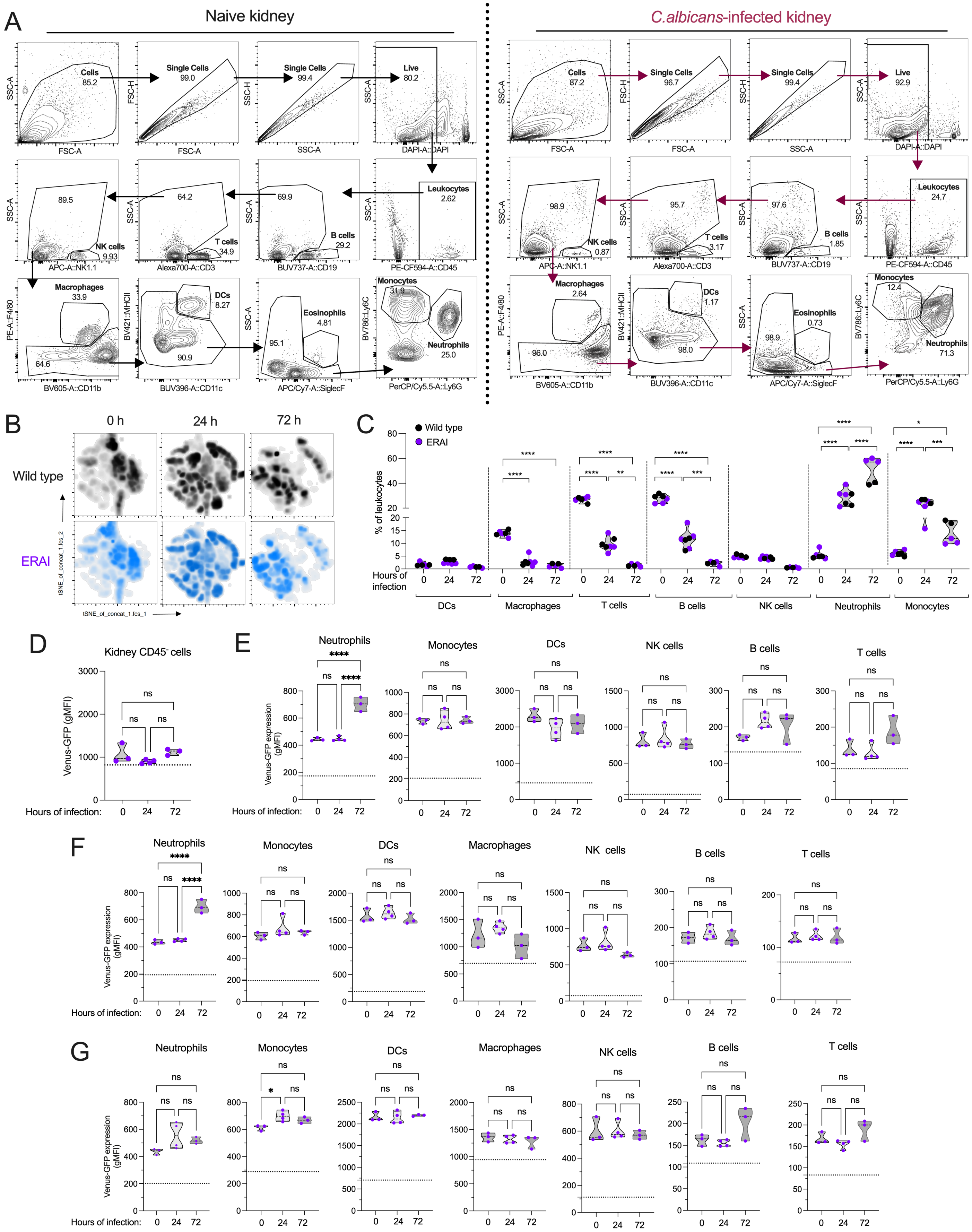

<u>Supplementary Figure 1.</u> Analysis of WT or ERAI mice with systemic candidiasis. (**A-F**) ERAI or wild type C57BL/6J mice (*n* = 4 per genotype per time point) were left untouched or injected i.v. with 10^5 *C. albicans* cells, and their kidneys, blood, spleen, and bone marrow were collected at the indicated time points. Single-cell suspensions were procured from various organs, as described in the methods, and stained with fluorescently-labeled antibodies specific for CD45, CD19, CD3, NK1.1, F4/80, CD11c, MHC-II, SiglecF, CD11b, Ly6C, Ly6G and DAPI. (**A**) Gating strategy used to analyze the kidney immune contexture in naïve or *C. albicans*-infected mice. FACS plots are representative of kidney analysis in an uninfected (naïve) mouse or in a mouse systemically infected with *C. albicans* for 72 h. (**B**) tSNE plots representing time-dependent changes in kidney total $CD45^+$ immune cells of ERAI or wild type mice. (**C**) Violin plots showing proportion of the indicated immune cell subsets within total $CD45^+$ leukocytes infiltrating the kidney at 0, 24, and 72 h after *C. albicans* infection. (**D-G**) Mean fluorescence intensity (gMFI) of Venus reporter expression in CD45-negative cells in the infected kidney. (**E-G**) Violin plots for gMFI of Venus reporter expression in the indicated immune cell types in blood (**D**), spleen (**E**), and bone marrow (**F**). (**D-G**) Dashed lines represent intrinsic autofluorescence in WT mice. (**C-G**) One-way ANOVA (Tukey's test). $^*P < 0.05$, $^{**}P < 0.005$, $^{***}P < 0.0005$, $^{****}P < 0.0001$, ns, not significant.

Supplementary Figure 2

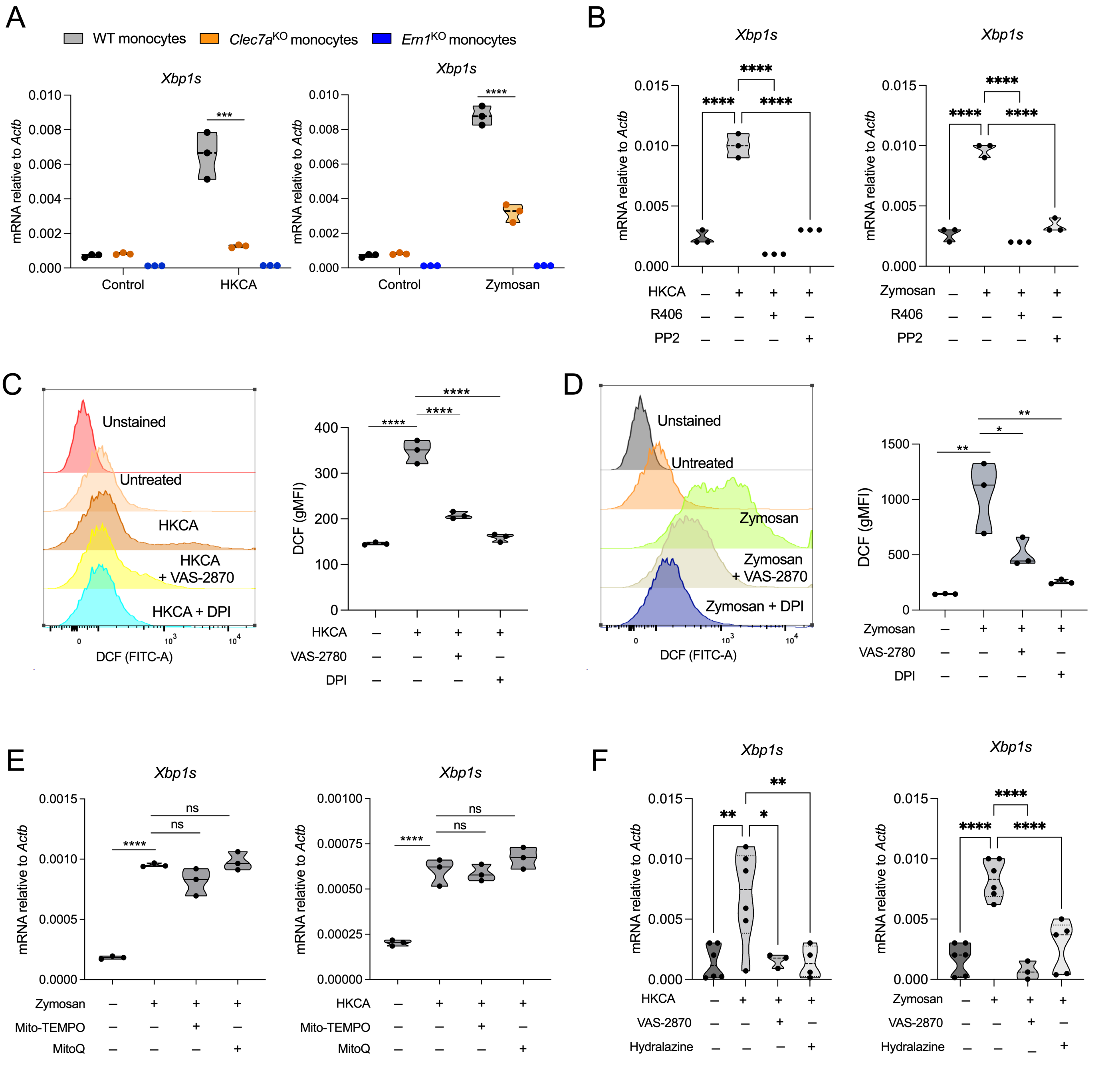

Supplementary Figure 2. The Dectin-1-Syk-NOX axis also mediates IRE1α activation in monocytes responding to zymosan or *C. albicans*. (**A**) Bone marrow-resident monocytes were isolated from mice of the indicated genotypes (n = 3 per genotype) and then stimulated for 6 h with HKCA (MOI=10) or zymosan (25 μg/ml). *Xbp1s* transcript levels were measured using quantitative RT-PCR. (**B**) Bone marrow-resident monocytes from WT C57BL/6J mice (n = 3) were pretreated for 1 h with vehicle control or the Syk inhibitor R406 (10 μM), and cells were then stimulated for 6 hours with HKCA (MOI=10) or zymosan (25 μg/ml). *Xbp1s*

transcript levels were measured using quantitative RT-PCR. **(C** and **D)** WT bone marrow neutrophils (*n* = 3 mice) were pretreated for 30 min with vehicle control or ROS inhibitors DPI (10 μM) or VAS-2870 (10 μM) and then stimulated with either **(C)** HKCA (MOI=10) or **(D)** zymosan (25 μg/ml) for 1 hour. ROS production was measured by flow cytometry as described in the methods. (**E**) WT bone marrow neutrophils (*n* = 3 mice) were pretreated for 30 min with vehicle control or mitochondrial ROS scavengers, Mito-TEMPO (10 μM) or MitoQ (2 μM), and then stimulated with either zymosan (25 μg/ml) or HKCA (MOI=10) for 6 hours. *Xbp1s* transcript levels were measured using quantitative RT-PCR. (**F**) Bone marrow-resident monocytes isolated from WT mice (*n* = 3-6 mice) were pretreated with VAS-2870 or hydralazine and then stimulated for 6 hours with HKCA (MOI=10) or zymosan (25 μg/ml). Representative violin plots are shown from at least 2 independent experiments with similar results. *Xbp1s* transcript levels were measured using quantitative RT-PCR. Data are shown as violin plots. One-way ANOVA (Tukey's test) was used for statistical analysis; **P* < 0.05, ***P* < 0.005, **** *P* <0.0001. gMFI, geometric mean fluorescence intensity. ns, not significant.

Supplementary Figure 3

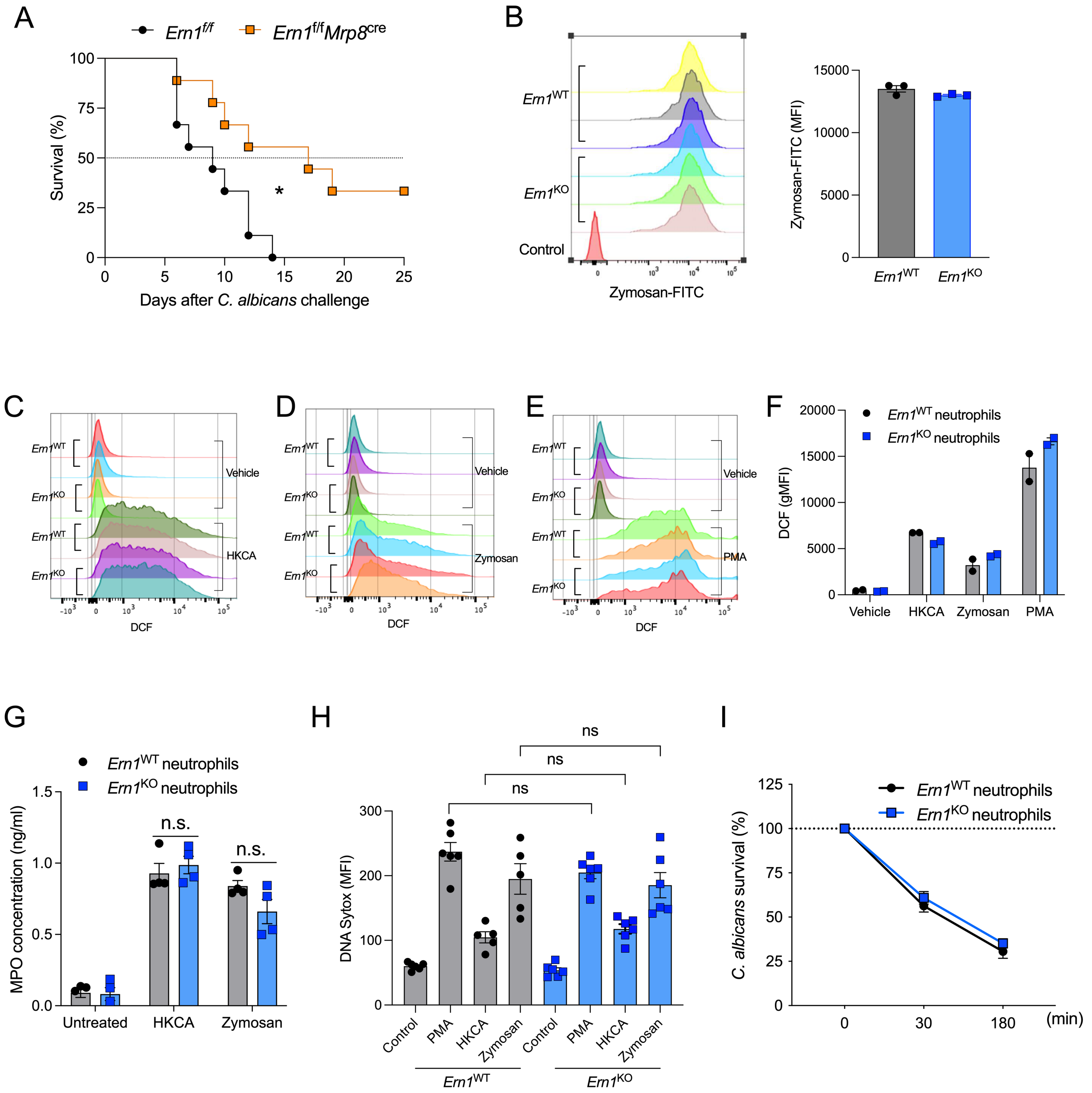

Supplementary Figure 3. Loss of IRE1α in neutrophils increases overall survival in mice with systemic candidiasis without altering their anti-*C. albicans* effector functions. (**A**) *Ern1*$^{f/f}$ (*n* = 9) or *Ern1*$^{f/f}$*Mrp8*^{cre} (*n* = 9) mice were infected i.v. with 10^5 *C. albicans* and overall host survival was monitored. (**B**) *Ern1*^{WT} or *Ern1*^{KO} neutrophils (*n* = 3 per genotype) were incubated with FITC-labeled zymosan for 30 min and their phagocytic capacity was assessed by FACS. (**C-F**) *Ern1*^{WT} or *Ern1*^{KO} neutrophils (*n* = 2 per genotype) were stimulated with (**C**) HKCA (MOI=5), (**D**) zymosan (25 μg/ml) or (**E**) PMA (50 nM) for 1 h. (**F**) ROS production was then quantified by FACS using the intensity of DCF signal generated. **(G)** *Ern1*^{WT} or *Ern1*^{KO} neutrophils (*n* = 4 per genotype) were

stimulated with HKCA (MOI=5) or zymosan (25 μg/ml) for 6 h and myeloperoxidase (MPO) production was measured in supernatants by ELISA. **(H)** *Ern1*^{WT} or *Ern1*^{KO} neutrophils (*n* = 5-6 per genotype) were stimulated with HKCA (MOI=20), zymosan (25 μg/ml) or PMA (100 nM) for 8 hours and DNA release was measured as a marker for NETosis using Sytox green. (**I**) *Ern1*^{WT} or *Ern1*^{KO} neutrophils (*n* = 4) were isolated and co-cultured with the yeast form of *C. albicans* for the indicated time points and CFUs were determined by serial dilutions on YPD agar. Percent survival was determined by normalization to *C. albicans* cultured without neutrophils. Data are shown as mean ± SEM. (**A**) Log-rank test **P*<0.05. (**G** and **H**) One-way ANOVA (Tukey's test) was used for statistical analysis; ns, not significant; MFI, mean fluorescence intensity; gMFI, geometric mean fluorescence intensity.

Supplementary Figure 4

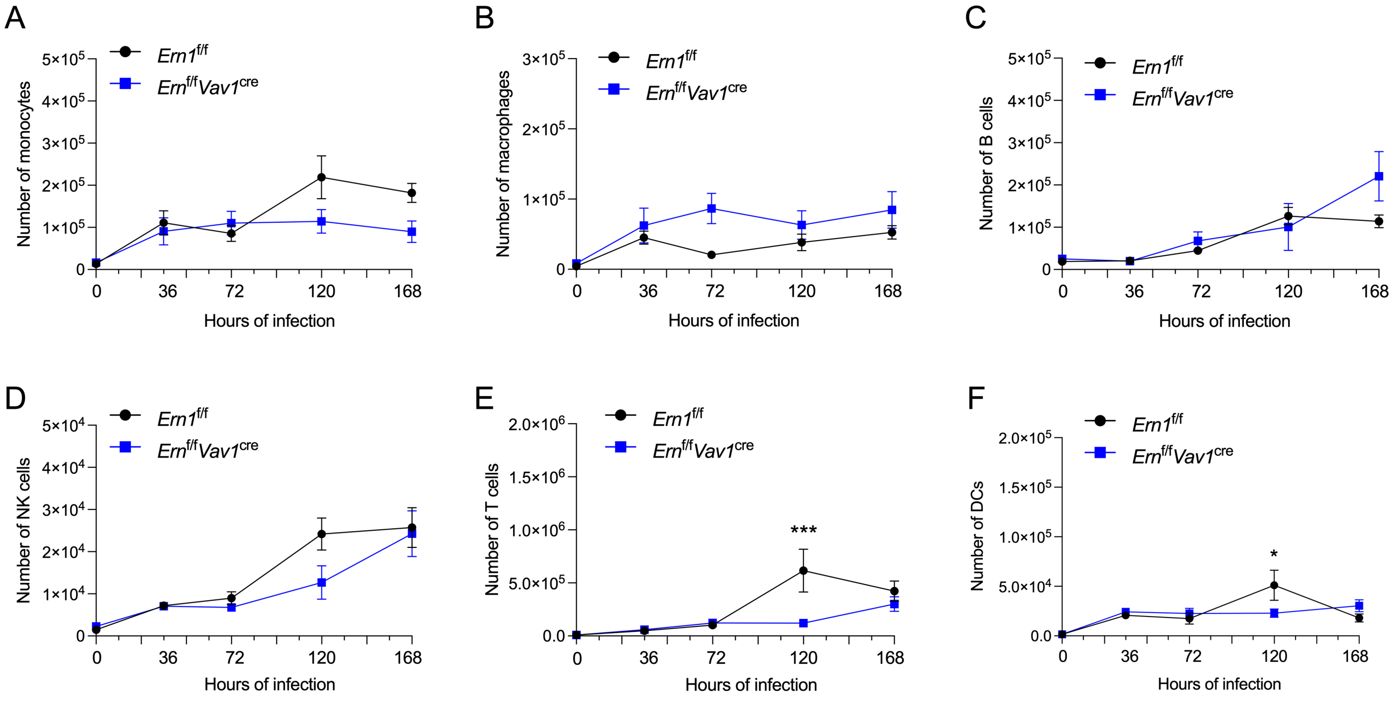

<u>Supplementary Figure 4.</u> Additional immune cells infiltrating the kidney during systemic *C. albicans*-infection. *Ern1*$^{f/f}$ or *Ern1*$^{f/f}$ *Vav1*^{cre} mice (n = 3-4 per genotype per time point) were infected i.v. with 10^5 *C. albicans* cells and the number of (**A**) monocytes, (**B**) macrophages, (**C**) B cells, (**D**) NK cells, (**E**) T cells, and (**F**) DCs in the kidney were determined by flow cytometry at the indicated times. Data are shown as mean ± SEM. Two-way ANOVA (Šídák's multiple comparisons test). *P < 0.05, ***P < 0.0005.

Supplementary Figure 5

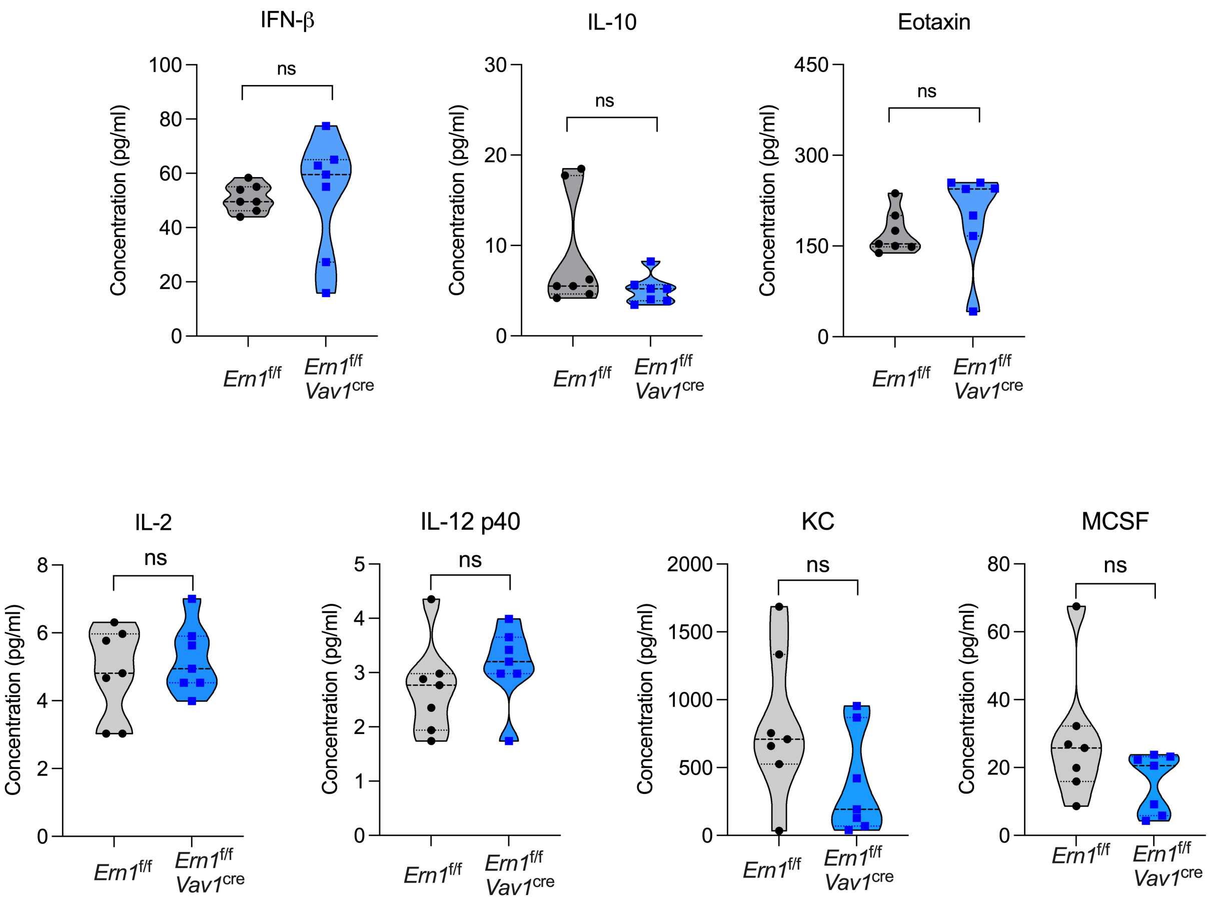

<u>Supplementary Figure 5.</u> Additional cytokines in kidney homogenates from *C. albicans*-infected mice. *Ern1*$^{f/f}$ (*n* = 7) or *Ern1*$^{f/f}$ *Vav1*^{cre} (*n* = 7) mice were infected i.v. with 10^5 *C. albicans* and expression of the indicated factors was determined by ELISA in total kidney homogenates 3 days post-infection. Data are shown as violin plots with dots representing independent mice. Two-tailed Student's *t*-test was used for statistical analysis. ns, not significant.

Supplementary Table 1.

Differentially expressed genes in kidney-infiltrating neutrophils and monocytes isolated from *Ern1*$^{f/f}$ vs. *Ern1*$^{f/f}$*Vav1*^{cre} mice systemically infected with *C. albicans* for 36 hours.

P.adj<0.05, Log2FoldChange >=1.0. UP, upregulated; DN, downregulated

Gene	DEG	Log2FoldChange	P.adj	Downstream pathways - Hallmark gene sets
Oas1g	UP	3.232091379	2.95E-12	
H2-DMb2	UP	3.203485546	4.15E-09	
Ly6a	UP	3.389266255	1.71E-08	
Iigp1	UP	3.981503671	4.58E-07	
Apol9a	UP	5.139129155	1.14E-06	
Gm4951	UP	5.215875903	1.88E-06	
Gbp8	UP	3.52817572	4.16E-06	
Ifi27l2a	UP	2.40344031	4.79E-06	
Slc4a8	UP	2.726860312	1.02E-05	
Oas1a	UP	1.306207703	1.46E-05	
Gm4841	UP	3.922026056	4.71E-05	
AC125149.3	UP	2.216308261	5.01E-05	
Hist3h2a	UP	1.358142636	5.82E-05	
Commd10	UP	2.500131721	6.78E-05	
Adi1	UP	1.446226755	0.000180868	
Casp12	UP	2.776045245	0.000180868	
Ccnd1	UP	2.076062651	0.000214936	
BC023105	UP	3.902767585	0.000309536	
Bckdhb	UP	1.63961766	0.000309536	
Smim24	UP	1.523782808	0.000309536	
Gbp4	UP	2.155434105	0.000401785	
Ramp1	UP	1.285300689	0.000698441	
Scimp	UP	2.049182673	0.000801816	
AC132444.2	UP	2.257859962	0.001080291	
AC132444.4	UP	2.257859962	0.001080291	
AC133103.6	UP	2.257859962	0.001080291	
Ubd	UP	4.344593784	0.001095723	
Cd200r4	UP	1.476408397	0.001110358	
Khk	UP	1.237857803	0.001376305	
Grap2	UP	3.108341882	0.001785902	
Fcgrt	UP	1.067513624	0.002470116	
Ifitm3	UP	1.159285309	0.003885756	
Nme3	UP	1.18402526	0.003969311	
Cryab	UP	2.75255852	0.004053168	
Tcea3	UP	1.406040878	0.004065582	
Camp	UP	4.701632212	0.007555553	
Hint2	UP	1.464080466	0.007566823	
Tmem205	UP	1.439318102	0.007767448	

Apoh	UP	2.740138305	0.011044951	
Sectm1b	UP	2.582254962	0.011044951	
Ccdc34	UP	1.75117771	0.011261551	
Ifit1bl1	UP	2.274692913	0.011963773	
H2-Eb1	UP	2.939587697	0.012318104	
Gbp6	UP	2.192091354	0.013063743	
Pigr	UP	3.93665134	0.015344319	
Epsti1	UP	1.007335245	0.017079724	
Nxn	UP	1.544503478	0.018479486	
Maf	UP	2.059623179	0.018857227	
Hoxa7	UP	2.701686827	0.019271808	
Gbp10	UP	3.507591017	0.0207083	
Apol7c	UP	4.05184554	0.021165601	
AC168977.1	UP	1.463842895	0.023255764	
Xaf1	UP	1.654432913	0.023426286	
Rnf227	UP	2.304538187	0.024141237	
Apol9b	UP	3.961197664	0.026003455	
Mitd1	UP	1.20634796	0.026290847	
Hdac11	UP	5.218413566	0.027243081	
AC132444.1	UP	2.081988305	0.028039066	
Ifi208	UP	1.890164083	0.028039066	
Rps27	UP	1.087105144	0.03078014	
Atp5l	UP	1.007489686	0.034475582	
Cpq	UP	1.287278066	0.034551724	
Dctpp1	UP	1.20302006	0.035038027	
C4b	UP	1.557325163	0.035226493	
Pla2g16	UP	1.903003856	0.035226493	
Ccdc107	UP	1.32751598	0.037582108	
Tgtp2	UP	1.562238402	0.037582108	
Clmn	UP	2.209363388	0.038025183	
H2-Aa	UP	2.556981724	0.040420453	
Cisd1	UP	1.134485956	0.043011749	
Gm16026	UP	1.665547866	0.044366098	
Timm10b	UP	1.155789109	0.04455419	
Ighm	UP	1.035307197	0.044576796	
Smim4	UP	1.202240535	0.046055339	
Mcts2	UP	2.539776615	0.046181067	
Cryl1	UP	1.129176672	0.047943986	
Rab13	UP	2.040784748	0.047943986	
Vill	UP	1.108867264	0.047943986	
Fau	UP	1.079821271	0.048564021	
Crim1	UP	2.256496654	0.049153842	
H2-Ab1	UP	2.400859347	0.049504026	
Ceacam1	DN	-1.988805987	4.15E-09	
Nr4a1	DN	-2.152063916	7.05E-09	HALLMARK_TNFA_SIGNALING_VIA_NFKB

Stk10	DN	-1.625835001	7.05E-09	
Fam20c	DN	-2.526752492	1.62E-07	
Tbc1d8	DN	-2.48423362	3.30E-07	
Tiam2	DN	-2.008482429	6.33E-06	
Adgb	DN	-4.213969269	8.69E-06	
Cxcl14	DN	-4.050168573	9.83E-06	
Gm38431	DN	-3.912335866	2.18E-05	
Dock5	DN	-1.776524228	6.42E-05	
Tbc1d2b	DN	-1.757772647	9.58E-05	
Zmiz1	DN	-1.09977427	0.00013969	
Itgb3	DN	-2.698024074	0.000180868	HALLMARK_INFLAMMATORY_RESPONSE, HALLMARK_IL6_JAK_STAT3_SIGNALING
Slc6a12	DN	-4.315717071	0.000180868	
Arg1	DN	-4.025086764	0.000198969	
Fkbp5	DN	-2.433406704	0.000198969	
Hic1	DN	-3.524529567	0.00025309	
Tnfrsf9	DN	-3.845073558	0.000309536	HALLMARK_TNFA_SIGNALING_VIA_NFKB, HALLMARK_INFLAMMATORY_RESPONSE, HALLMARK_IL2_STAT5_SIGNALING
Plagl2	DN	-1.769699281	0.000330641	
Fabp4	DN	-3.261477057	0.000357474	
Tg	DN	-3.012118635	0.000357474	
Itgax	DN	-2.511488968	0.000485695	
Naip1	DN	-4.829189672	0.000511224	
Ikbke	DN	-2.204227521	0.000524476	
Tmem119	DN	-3.021863873	0.000620004	
Tnip1	DN	-1.658014742	0.000629866	HALLMARK_TNFA_SIGNALING_VIA_NFKB
Nos2	DN	-2.93310251	0.000716616	
Ace	DN	-1.87376367	0.00080052	
Adam17	DN	-1.54609317	0.001080291	
Nfatc3	DN	-1.296041476	0.001080291	
Sema4d	DN	-1.857170531	0.001096826	HALLMARK_INFLAMMATORY_RESPONSE
Itgb1	DN	-1.404221175	0.001141832	
Treml4	DN	-2.172595734	0.001173512	
Nfkb2	DN	-1.409977429	0.001184046	HALLMARK_TNFA_SIGNALING_VIA_NFKB
Nfe2l1	DN	-1.305218519	0.001232878	
St6gal1	DN	-2.715715758	0.001246543	
Tnfrsf1b	DN	-1.379691198	0.001376305	HALLMARK_INFLAMMATORY_RESPONSE, HALLMARK_IL2_STAT5_SIGNALING, HALLMARK_IL6_JAK_STAT3_SIGNALING
Ibtk	DN	-1.575353545	0.001784011	
Rap1gds1	DN	-1.009878284	0.001900872	
Col5a1	DN	-4.065328662	0.002710904	
Ifnar1	DN	-1.310049699	0.002710904	HALLMARK_INFLAMMATORY_RESPONSE, HALLMARK_IL6_JAK_STAT3_SIGNALING
Ptprj	DN	-1.395249055	0.003120474	

Adgre4	DN	-2.296789497	0.003525844	
Malt1	DN	-1.283531565	0.003758597	
Slc7a2	DN	-3.428305246	0.004210174	HALLMARK_INFLAMMATORY_RESPONSE
Il4i1	DN	-2.093601792	0.004431651	
Acp5	DN	-1.396752531	0.004453897	
Cdc42ep4	DN	-1.345361876	0.004623762	
Jchain	DN	-4.561664262	0.004762463	
Pdgfb	DN	-2.603551704	0.005190962	
Myo1c	DN	-1.527898096	0.00580679	HALLMARK_IL2_STAT5_SIGNALING
Ltbp3	DN	-4.080768727	0.006122409	
Has1	DN	-3.712505146	0.006393246	
Creb3l2	DN	-2.741073315	0.006400908	
Sacm1l	DN	-1.325465789	0.006400908	
Kdm5c	DN	-1.342010108	0.010276852	
Adora2a	DN	-2.246079757	0.011152543	
P4ha1	DN	-1.696623023	0.011152543	HALLMARK_IL2_STAT5_SIGNALING
Tmc6	DN	-1.164999867	0.011152543	
Gpr84	DN	-1.714093539	0.011963773	
Spata13	DN	-1.963945301	0.011963773	
Ndufaf7	DN	-1.086323477	0.012305316	
Gm21985	DN	-7.189605336	0.012318104	
Ero1l	DN	-3.074328375	0.012817858	
Shc1	DN	-1.03335912	0.013307052	
Wnt6	DN	-2.085610045	0.013307052	
Orai2	DN	-1.013940594	0.013928597	
Tmc8	DN	-1.464213739	0.014054157	
Bhlhe40	DN	-2.382643831	0.014752568	HALLMARK_TNFA_SIGNALING_VIA_NFKB, HALLMARK_IL2_STAT5_SIGNALING
Cyth3	DN	-1.970592431	0.015636755	
Pan2	DN	-1.279272234	0.017079724	
Nfkbie	DN	-1.231331098	0.019271808	HALLMARK_TNFA_SIGNALING_VIA_NFKB
Plekhm3	DN	-1.110640588	0.019439476	
Smpdl3b	DN	-1.896573552	0.020132829	
Spg11	DN	-1.506075171	0.020543885	
Adam10	DN	-1.278695278	0.0207083	
Fam135a	DN	-3.138066263	0.021080179	
Arfgef2	DN	-1.452255668	0.021803462	
Kpna1	DN	-1.045169458	0.021993366	
C3	DN	-1.37115169	0.022220393	
Abca1	DN	-2.579011637	0.022729768	HALLMARK_TNFA_SIGNALING_VIA_NFKB, HALLMARK_INFLAMMATORY_RESPONSE
Plpp5	DN	-1.702498899	0.023431519	
Ulbp1	DN	-3.167163779	0.024141237	
Elf4	DN	-1.188220314	0.024988497	
Tgm3	DN	-2.440030591	0.025067011	

Acp2	DN	-1.943235457	0.026662889	
Rps6ka4	DN	-1.235922004	0.027779287	
Gys1	DN	-2.385319182	0.028039066	
Il10	DN	-3.732763622	0.028039066	HALLMARK_INFLAMMATORY_RESPONSE, HALLMARK_IL2_STAT5_SIGNALING
S1pr5	DN	-2.030749978	0.028150631	
Sec24d	DN	-2.764348104	0.028986659	
Tbc1d9b	DN	-1.111551993	0.03078014	
Ubqln1	DN	-1.12434983	0.03078014	
Frs2	DN	-1.280505667	0.032400753	
Jag1	DN	-2.304506625	0.032400753	HALLMARK_TNFA_SIGNALING_VIA_NFKB
Cd300e	DN	-2.518290701	0.033139761	
Tank	DN	-1.294686928	0.03383381	HALLMARK_TNFA_SIGNALING_VIA_NFKB
Eno3	DN	-1.869092324	0.035226493	HALLMARK_IL2_STAT5_SIGNALING
Dennd4b	DN	-1.146758397	0.035383651	
Itch	DN	-1.085504096	0.035383651	
Tnfaip3	DN	-2.247852235	0.035579275	HALLMARK_TNFA_SIGNALING_VIA_NFKB
Hip1	DN	-1.142316141	0.037582108	
Pygo2	DN	-1.048828733	0.037582108	
Jade3	DN	-2.421967462	0.039336806	
Phtf2	DN	-1.953464473	0.044214599	HALLMARK_IL2_STAT5_SIGNALING
Cnot1	DN	-1.318001649	0.046407323	
Dhx38	DN	-1.22416948	0.046538442	
Irak3	DN	-2.166800432	0.046760382	
Pdpn	DN	-2.45034046	0.048239154	HALLMARK_INFLAMMATORY_RESPONSE

Supplementary Table 2. Primers used in this study

Species	Transcript	Primer direction	Sequence 5'-3'	Purpose
mouse	*Xbp1*	Forward	ACACGCTTGGGAATGGACAC	Splicing assay
		Reverse	CCATGGGAAGATGTTCTGGG	
mouse	*Actb*	Forward	CTCAGGAGGAGCAATGATCTTGAT	RT-qPCR
		Reverse	TACCACCATGTACCCAGGCA	
mouse	*Xbp1s*	Forward	AAGAACACGCTTGGGAATGG	RT-qPCR
		Reverse	CTGCACCTGCTGCGGAC	
mouse	*Ddit3*	Forward	GTCCCTAGCTTGGCTGACAGA	RT-qPCR
		Reverse	TGGAGAGCGAGGGCTTTG	
mouse	*Hspa5/BiP*	Forward	TCATCGGACGCACTTGGAA	RT-qPCR
		Reverse	CAACCACCTTGAATGGCAAGA	
mouse	*Dnajb9/ERdj4*	Forward	TAAAAGCCCTGATGCTGAAGC	RT-qPCR
		Reverse	TCCGACTATTGGCATCCGA	
mouse	*Sec61a1*	Forward	CTATTTCCAGGGCTTCCGAGT	RT-qPCR
		Reverse	AGGTGTTGTACTGGCCTCGGT	
mouse	*Atf4*	Forward	GAGCTTCCTGAACAGCGAAGTG	RT-qPCR
		Reverse	TGGCCACCTCCAGATAGTCATC	
mouse	*Erp44*	Forward	GCTGAAACGACACCAGTCAG	RT-qPCR
		Reverse	CAGATGCTCCTTGCTGCTC	
mouse	*Rpn1*	Forward	GTTTCCACAACGACCGAGAT	RT-qPCR
		Reverse	CCTAGGCGTGCAGATAAAGG	
mouse	*HGSNAT*	Forward	CTGATGACTGTTACCAATGCACC	RT-qPCR
		Reverse	GCACCAAAAGGGAATAGTTTCCA	
mouse	*Tapbp*	Forward	GGAGGGTGTCTACCTGGCTA	RT-qPCR
		Reverse	AACGGGTGCTGGTGTTAGAG	
mouse	*Bloc1s1*	Forward	GAAGCGTTGGTGGATCACCT	RT-qPCR
		Reverse	TCACCTCATGGTCCAGCTTTC	

mouse	*Il6*	Forward	GAACAACGATGATGCACTTGC	RT-qPCR
		Reverse	TCCAGGTAGCTATGGTACTCC	
mouse	*Tnf*	Forward	AATGGCCTCCCTCTCATCAGTT	RT-qPCR
		Reverse	CCACTTGGTGGTTTGCTACGA	
mouse	*Il1b*	Forward	CTCCACCTCAATGGACAGAA	RT-qPCR
		Reverse	GCCGTCTTTCATTACACAGG	
mouse	*Ptgs2*	Forward	TGGGTGTGAAGGGAAATAAGGAG	RT-qPCR
		Reverse	ATTTGAGCCTTGGGGGTCAG	
